# Testing unit root non-stationarity in the presence of missing data in univariate time series of mobile health studies

**DOI:** 10.1093/jrsssc/qlae010

**Published:** 2024-02-29

**Authors:** Charlotte Fowler, Xiaoxuan Cai, Justin T Baker, Jukka-Pekka Onnela, Linda Valeri

**Affiliations:** Department of Biostatistics, Mailman School of Public Health, Columbia University, New York, NY, USA; Department of Statistics, The Ohio State University, Columbus, OH, USA; Institute for Technology in Psychiatry, McLean Hospital, Harvard Medical School, Belmont, MA, USA; Department of Biostatistics, Harvard TH Chan School of Public Health, Harvard University, Boston, MA, USA; Department of Biostatistics, Mailman School of Public Health, Columbia University, New York, NY, USA

**Keywords:** missing data, mobile health, stationarity, time-series analysis

## Abstract

The use of digital devices to collect data in mobile health studies introduces a novel application of time series methods, with the constraint of potential data missing at random or missing not at random (MNAR). In time-series analysis, testing for stationarity is an important preliminary step to inform appropriate subsequent analyses. The Dickey–Fuller test evaluates the null hypothesis of unit root non-stationarity, under no missing data. Beyond recommendations under data missing completely at random for complete case analysis or last observation carry forward imputation, researchers have not extended unit root non-stationarity testing to more complex missing data mechanisms. Multiple imputation with chained equations, Kalman smoothing imputation, and linear interpolation have also been used for time-series data, however such methods impose constraints on the autocorrelation structure and impact unit root testing. We propose maximum likelihood estimation and multiple imputation using state space model approaches to adapt the augmented Dickey–Fuller test to a context with missing data. We further develop sensitivity analyses to examine the impact of MNAR data. We evaluate the performance of existing and proposed methods across missing mechanisms in extensive simulations and in their application to a multi-year smartphone study of bipolar patients.

## 1 Introduction

Assessing the stationarity of a univariate time series has long been a question of interest to evaluate data assumptions and inform subsequent analyses. However, solutions for testing for stationarity have been largely developed for the field of economics, where the data are typically recorded as a fully observed time series. The increase in the prevalence of time-series data across disciplines (e.g. social science, political science, and psychiatry) introduces new challenges for existing time-series analysis methods.

One growing source of time-series data is personal digital devices. This technology is increasingly employed to collect information, particularly in the field of health research (Aledavood et al., 2017; Silva et al., 2015). It can record participants’ real-time exposures and outcomes, with the potential for many observations per second (Vaidya et al., 2013; Torous et al., 2016, 2018). Mobile health (mHealth) studies for example utilize individuals’ smartphones and wearable devices to collect information on the participants daily activities and well-being (Aledavood et al., 2017; Mandel et al., 2021). As smartphones become essential tools for day-to-day life, mHealth study designs will become ever-more prevalent, and necessitate the development of new statistical methods to handle the particular challenges that mobile health data can introduce. Mobile health data have two forms: active and passive. Active data are collected directly from the participant, such as daily survey responses provided by participants. Passive data are observed with no action required from the participant, such as Global Positioning System (GPS) records, accelerometer data, and phone and text logs. Both types of data may suffer from pervasive missingness resulting from technological errors, inactive devices, or participants being unable, unwilling, or uninterested in engaging with the software. The bipolar longitudinal study (BLS) is an mHealth cohort study of 74 individuals with Bipolar I or II disorder, schizophrenia, or schizoaffective disorder followed for up to five years using the Beiwe application (Barnett & Onnela, 2020; Huang & Onnela, 2019; Onnela et al., 2021) on the participant’s smartphone. It was configured in the study to record basic information of the participant’s anonymized phone calls and text messages, their accelerometer and GPS data, and daily responses to a 31 item survey including questions on mood, sleep, social interaction, physical activity, and other health conditions (Cai et al., 2022). The study provides researchers with the possibility of identifying the causal effects of at home interventions such as increased social or physical activity on improving an individual’s mood and symptoms. However, the validity of such analyses will be dependent on modelling assumptions, including stationarity and the hypothesized missing mechanism. In the bipolar longitudinal study, we observe missing data rates for the daily survey varying from 2% to over 90%. Missingness is also pervasive in passive data and could result from a variety of factors including glitches, noncompliance, or failure to update software. For example, for one participant in the BLS study we fail to observe any text data for over a four-month gap, or 16% of the follow up their period. Preliminary results in the bipolar longitudinal study also indicate the presence of non-stationarity among several participants’ time-series data (Cai et al., 2022).

In time-series analysis, stationarity is defined as constant mean and variance across time (Metcalfe & Cowpertwait, 2009). There are many causes of non-stationarity such as change points and unstable variance, but the most commonly evaluated is unit root. Unit root non-stationarity is characterized by an autocorrelation of one between the current state and lagged values. A common example of a unit root time series is a random walk. Most simple modelling methods for longitudinal data assume that no unit roots are present, as a unit root will introduce undesirable properties (Choi, 2015). This is true whether data are analysed across individuals, or an N-of-1 approach is employed (Kwasnicka & Naughton, 2020; Vieira et al., 2017). Specifically, we see that when a unit root is present the variance of the process will diverge to infinity as the follow up length becomes infinite, the time series will have no tendency to return to its average value, and the autocorrelation between two time points will not decrease regardless of the distance separating them (Choi, 2015). These properties introduce challenges to estimation and result in reduced predictive ability (Choi, 2015; Dickey et al., 1986). Additionally, researchers have repeatedly shown that when traditional regression methods are inappropriately applied to a time series with unit root, results may be spurious, including detection of falsely significant relationships between independent time series and inconsistent estimators (Dickey et al., 1986; Granger & Newbold, 2014; Plosser & Schwert, 1978).

Due to limited follow up in many mobile health studies, researchers often aggregate across participants and ignore fine grain data (Aledavood et al., 2017; Luckett et al., 2019; Tewari & Murphy, 2017). Specifically, we see that tools such as linear mixed models are overwhelmingly used (Chaibub Neto et al., 2016; Luckett et al., 2019; Tewari & Murphy, 2017). However, the validity of these regression methods still depends on stationarity and the absence of unit roots. As mobile health studies become more common and are designed to record denser information over a longer period of time, researchers will have a greater ability and responsibility to thoroughly investigate the assumption of stationarity prior to selecting the appropriate statistical method for analysis, regardless of their choice to aggregate across individuals for analysis or treat data using an N-of-1 approach. Indeed, initial results using mobile health data with a follow up period of over four years and modelling methods that allow for non-stationarity indicate that unit-root is likely present, and in fact identify an intercept term that acts as a random walk (Cai et al., 2022). These results demonstrate that unit root processes are not just an economic phenomenon and are likely present yet undetected in health data. We hypothesize that unit root testing is typically omitted from mHealth studies since the conventional method to do so, Dickey–Fuller (DF) test, was developed for an economic setting where data are fully observed (Metcalfe & Cowpertwait, 2009). Notably, usage of this test requires complete data.

There are three classifications for missing data to determine its relative impact on analyses and validity: missing completely at random (MCAR), missing at random (MAR), and missing not at random (MNAR) (Little & Rubin, 2019). When data are missing completely at random, the likelihood of a data point being observed is not influenced by any observed or unobserved data; this is the optimal scenario. In the case of daily surveys from mHealth studies, this could be conceived as a participant failing to respond to survey questions at random a few days a week. Alternatively, when data are MAR, the probability of missingness is only affected by observed variables. An example of missing at random in the mHealth context is loss of interest in study engagement, where missing rates of survey responses increase with time in the study. Lastly, the most challenging mechanism is if data are missing not at random, and the probability of missing data is influenced by unobserved information. For mobile health, this might be represented by an individual not responding to questions on their mood, due to an unobserved depressive state. Because it is hypothesized that mHealth data are often missing at random or missing not at random (Goldberg et al., 2021), time-series methods must be adapted to these contexts.

The existing missing data methods used in time-series analysis impose strong assumptions about unit root, and thus may have impacts on validity when used with the DF test. Basic methods such as mean imputation and last observation carry forward (LOCF) are known to bias estimates and distort variance even when data are MCAR (Little & Rubin, 2019). Linear and spline interpolation have been shown to produce accurate estimates when used for imputation in covariates (Skjelbred & Kong, 2019; Terry et al., 1986) but likely bias autocorrelation estimates due to their imposition on the autocorrelation structure. Wijesekara and Liyanage (2020) showed under MCAR that Kalman smoothing (Moritz & Bartz-Beielstein, 2017) achieves high accuracy when imputing values in a univariate time series, however the method has not been evaluated on other missing mechanisms. Commonly used multiple imputation methods developed for non-longitudinal data, such as multiple imputation with chained equations fail to account for the correlation between the lagged and current value when imputing variance (Azur et al., 2011; Little & Rubin, 2019), and thus may provide unreliable results. Lastly, complete case analysis which simply drops all unobserved time points and thus disrupts the time sequence may be appropriate when data are MCAR (Shin & Sarkar, 1996). If the time series has unit root, the autocorrelation will be maintained, while if the autocorrelation is less than one, the autocorrelation will be underestimated.

The existing research on stationarity testing and autocorrelation estimation with incomplete data is sparse, and relies on strong assumptions about the missingness mechanism. Little and Rubin (2019) proposed an expectation-maximization (EM) algorithm for autocorrelation estimation for an AR(1) time series, but condition on stationarity. Shin and Sarkar (1994, 1996) demonstrated that if the time series follows an AR or ARMA structure and data are MCAR, then complete case analysis or last observation carry forward are sufficient methods to be used prior to the augmented Dickey–Fuller test. Park et al. (2005) developed a Bayesian test for incomplete data that tests multiple hypotheses of asymmetry and unit root non-stationarity. However, there is a need for the investigation of the impact of different missing mechanisms and rates on unit root testing, and a consideration of more sophisticated missing data methods for the DF test.

We first propose using imputations from a state space model with multiple imputation (SSMimpute) (Cai et al., 2022) for unit root testing with the DF test. While this method is shown to produce non-biased coefficients for covariate estimates with multivariate time series analysis, it has not been tested with a univariate time series and estimation of the lagged value coefficient. Under the assumption of a single lag order and normally distributed errors, we additionally propose two likelihood maximization approaches to adapt the DF test to the context of missing data. The first uses mathematical optimization to maximize the observed data likelihood, and applies the DF test using the obtained estimates. The second incorporates an EM algorithm to recursively impute missing data and estimate the autocorrelation and variance, and runs the DF test on the final imputed time series. We additionally introduce sensitivity analyses with a *δ* adjustment for the state space model with multiple imputation and maximum likelihood approach using an EM algorithm to consider the ramifications of MNAR data on unit root testing. The remainder of this paper will first review unit root testing without missing data, then expand these methods to the context of MCAR and MAR data, and lastly introduce sensitivity analyses for MNAR data. We will use simulation and application to BLS mobile health data to review validity and performance of proposed methods.

## 2 Testing unit root without missing data

Let *t* represent time point from t=0,1,…,T. Then assuming lag order of one, we can define the time series $Y_{t}$ such that $y_{t}=\rho y_{t-1}+\epsilon_{t}$, where $E(\epsilon_{t})=0$, $Var(\epsilon_{t})=\sigma^{2}$, and $\epsilon_{t}$ are independently and identically distributed. We state that time series *y* has unit root if ρ=1. If this is true, the process is known as a random walk, and as T→∞, the variance of the time series will diverge to infinity (Metcalfe & Cowpertwait, 2009). This renders various statistical assumptions invalid, such as those needed to apply standard longitudinal analysis methods including linear mixed models. Notably, preliminary analysis of the bipolar longitudinal study has identified that some participants’ response patterns appear to follow a random walk, demonstrating the importance of testing for unit root in mHealth data (Cai et al., 2022). The DF test was developed to test the null hypothesis: $H_{0}\;:\;\rho =1$ against the alternative that $H_{1}\;:\;\rho <1$ (Dickey & Fuller, 1979). The test employs ordinary least square methods regressing $Y_{t}$ by $Y_{t-1}$ to estimate $\hat{\rho }$ and ${\hat{\sigma }}^{2}$ such that test statistic is defined as:


(1) $$DF_{\hat{\rho }}=\frac{\hat{\rho }-1}{SE(\hat{\rho })}=\left(\frac{\sum y_{t}y_{t-1}}{\sum y_{t-1}^{2}}-1\right)/\sqrt{\frac{T{\hat{\sigma }}^{2}}{T\sum y_{t-1}^{2}-(\sum y_{t-1}{)}^{2}}}.$$

Under the null hypothesis, the distribution of the test statistic has a non-standard form due to the properties of a unit root process (Dickey & Fuller, 1979). For testing purposes, *p*-values and rejection regions are generated through interpolation, using a table of distribution quantiles which were obtained via simulation (Dickey, 1976; Dickey & Fuller, 1979). Assuming $\epsilon_{t}$ are distributed normally, we note that $Y_{t}|Y_{t-1}∼N(\rho y_{t-1},\sigma^{2})$. We can thus solve for the joint log-likelihood of $\rho ,\sigma^{2}$ conditional on $y_{1},\ldots ,y_{T}$ as:


(2) $$\ell (\rho ,\sigma^{2}|y_{1},\ldots ,y_{T})=\sum_{t=1}^{T}\log\;f(y_{t}|y_{t-1})=-T/2\log\;(2\pi \sigma^{2})-\frac{1}{2\sigma^{2}}\sum_{t=1}^{T}(y_{t}-\rho y_{t-1}{)}^{2}.$$

## 3 Testing unit root in the presence of MCAR or MAR missing data

For t=1,…,T, let $r_{t}$ serve as an indicator of whether $y_{t}$ is observed, and $p_{t}$ represent the probability of observing $y_{t}$, i.e. $p_{t}=P(r_{t}=1)$. If data are missing completely at random, we assume that $p_{t}$ is independent of $Y_{t},t$, and any other observed or unobserved variables, for t=1,…,T. When the time series is missing at random, $p_{t}$ is dependent only on observed variables. In the case of a univariate time series under MAR, we will assume that the probability of observing a time point *t* is dependent on only observed covariates $X_{t}$ including time *t* and previously observed non-missing $y_{t}$, such that for some function *g*, $p_{t}=g(X_{t})$. We let $t_{1},\ldots ,t_{n}$ represent the index of times with non-missing observations, such that $y_{t_{1}},y_{t_{2}},\ldots ,y_{t_{n}}$ represent all observed time series values, with *n* total non-missing observations, and n≤T. Note that as shown in Bertin et al. (2011), we can solve that the distribution of $y_{t_{k}}$ conditional on the previously observed point $y_{t_{k-1}}$ is normal, such that


(3) $$Y_{t_{k}}|Y_{t_{k}-1}∼N(\rho^{t_{k}-t_{k-1}}y_{t_{k}-1},\sigma^{2}V_{k}(\rho )),\text{ with }V_{k}(\rho )=\sum_{\;j=1}^{t_{k}-t_{k-1}}\rho^{2(j-1)}.$$

We can now adapt the complete time series log-likelihood from Eq. (2) for observed data:


(4) $$\begin{array}{rl} \ell (\rho ,\sigma^{2}|y_{t_{1}},\ldots ,y_{t_{n}}) & =\sum_{t=1}^{T}\left(r_{t}\log\;p_{t}+(1-r_{t})\log\;(1-p_{t})\right)+\sum_{k=1}^{n}\log\;f(y_{t_{k}}|y_{t_{k-1}})\\ & =\sum_{t=1}^{T}\left(r_{t}\log\;p_{t}+(1-r_{t})\log\;(1-p_{t})\right)-n/2\log\;(2\pi \sigma^{2})\\ & \;-\sum_{k=1}^{n}\left(\frac{1}{2}\log\;V_{k}(\rho )-\frac{y_{t_{k}}-\rho^{(t_{k}-t_{k-1}y_{t_{k-1}}{)}^{2}}}{2\sigma^{2}V_{k}(\rho )}\right). \end{array}$$

Note that when $p_{t}$ is independent of *ρ* and *σ*, the likelihood can be maximized with respect to these parameters by disregarding the first term of the log-likelihood. This condition holds when data is MCAR or MAR.

### 3.1 Maximum likelihood estimation with numeric optimization

Using likelihood from Eq. (4), where we assume $\epsilon_{t}$ is distributed normally, even if we assume data are MCAR or MAR and ignore the first term, we are unable to solve for an algebraic maximum. Instead, we suggest using numerical optimization, specifically the Nelder–Mead method (Nelder & Mead, 1965) to maximize the function with respect to $\sigma^{2}$ and *ρ*. We calculate initial estimates of *ρ* and *σ* using only time points where $y_{t}$ and $y_{t-1}$ are observed, such that


(5) $${\hat{\rho }}_{\left(0\right)}=\frac{\sum_{t=1}^{T}r_{t}r_{t-1}y_{t}y_{t-1}}{\sum_{t=1}^{T}r_{t}r_{t-1}(y_{t-1}{)}^{2}}\;\text{and}\;{\hat{\sigma }}_{\left(0\right)}^{2}=\frac{\sum_{t=1}^{T}r_{t}r_{t-1}(y_{t}-y_{t-1}{)}^{2}}{\sum_{t=1}^{T}r_{t}r_{t-1}}.$$

To implement the DF test using estimates generated from numerical maximization ( $\hat{\rho },\hat{\sigma^{2}}$), we calculate a conservative new test statistic as:


(6) $$DF_{\hat{\rho },c}=\frac{\hat{\rho }-1}{SE(\hat{\rho })}=(\hat{\rho }-1)/\sqrt{\frac{n{\hat{\sigma }}^{2}}{n\sum_{k=1}^{n-1}y_{t_{k}}-{\left(\sum_{k=1}^{n-1}y_{t_{k}}\right)}^{2}}}.$$

We will refer to this method as maximum likelihood estimation by numerical optimization, conservative (MLEN). This statistic fails to leverage on the longer observation period, and treats the follow up time as strictly the number of non-missing time points. For time series with severe missing rates, or shorter follow up, the conservative nature of this test statistic becomes a limitation for its power. To address this issue, we additionally consider a scaled version of the above statistic, where we leverage on the unobserved time points, by calculating $DF_{\hat{\rho },s}=\frac{T}{n}DF_{\hat{\rho },c}$. We will refer to this method as maximum likelihood estimation by numerical optimization, scaled (MLENS).

When assumptions of normality and lag order of one are satisfied, the algorithm benefits from exact model specification, allowing for accurate results with computational ease.

### 3.2 Maximum likelihood estimation with expectation maximization algorithm

We additionally consider a maximum likelihood estimation approach using an iterative expectation maximization algorithm (MLEEM). We calculate initial estimates for the parameters as in Eq. (5). The algorithm then recursively imputes missing observations, and updates parameters until convergence. In iteration *j*, we impute missing observations chronologically such that for unobserved $y_{t}$, ${\hat{y}}_{t}^{\left(j\right)}={\hat{\rho }}_{\left(j-1\right)}{\hat{y}}_{t-1}^{\left(j\right)}$. In the M step, we maximize the likelihood using the imputed time series, which is equivalent to ordinary least squares estimation when regressing $Y_{t}$ by $Y_{t-1}$. Thus estimates are updated in iteration *j* such that


$${\hat{\rho }}_{\left(j\right)}=\frac{\sum {\hat{y}}_{t}^{\left(j\right)}{\hat{y}}_{t-1}^{\left(j\right)}}{\sum {\left({\hat{y}}_{t-1}^{\left(j\right)}\right)}^{2}}\;\text{and}\;{\hat{\sigma }}_{\left(j\right)}^{2}=\frac{\sum_{t=1}^{T}{\left({\hat{y}}_{t}^{\left(j\right)}-{\hat{y}}_{t-1}^{\left(j\right)}\right)}^{2}}{T}.$$

Similarly to the numerical optimization method, the EM approach for maximum likelihood estimation benefits from a exact model specification, and computational efficiency when assumptions hold. It additionally allows for direct calculation of the DF test statistic from the fully imputed time series, avoiding issues of reduced power encountered by numerical optimization without imputation.

### 3.3 State space model with multiple imputation

We lastly propose a more flexible imputation method to be used prior to unit root testing. The state space model with multiple imputation (SSMimpute) does not assume a single order lag structure, and in applications has been demonstrated to perform well with continuous data that is not normally distributed (Cai et al., 2022). Assuming *q* lag order, the state space model is fit by regressing $Y_{t}$ by its lagged values, $Y_{t-q},\ldots ,Y_{t-1}$. First, we generate initial estimates for the missing values of $Y_{t}$ using Kalman filtering, and impute these values in the lagged regressors, $Y_{t-q},\ldots ,Y_{t-1}$, but not $Y_{t}$. The algorithm then recursively until convergence estimates the coefficients of the regressors and the variance by maximizing the likelihood and calculating new imputations from the posterior to update missing values of $Y_{t-q},\ldots ,Y_{t-1}$.

Specifically, to maximize the likelihood, we employ a linear state space model, such that if $Y_{t}$ is expressed as $Y_{t}=\beta_{0_{t}}+\rho_{1,t}Y_{t-1}+\cdots +\rho_{q,t}Y_{t-q}+\epsilon_{t}$ with $\epsilon_{t}∼N(0,\sigma )$, we re-write $Y_{t}=F_{t}\theta_{t}+\epsilon_{t}$, where $F=(1,Y_{t-1},\ldots ,Y_{t-q})$ and $\theta_{t}$ denotes the state vector that encompasses the intercept and coefficients of the lagged regressors ( $\theta_{t}=(\beta_{0,t},\rho_{1,t},\ldots ,\rho_{q,t}{)}^{T}$). To fit the state space model, we additionally assume that $\theta_{t}$ is a Markov process, meaning that $\theta_{t}⊥\;\;\;⊥\theta_{s}|\theta_{t-1}$ for any s<t, and that $\theta_{t}=G_{t}\theta_{t-1}+w_{t}$ with $w_{t}∼N_{d}(0,W_{t})$, where $G_{t}$ denotes the state transition matrix, and *d* represents the dimension of *θ*. In our case d=q+1. Using Kalman smoothing and filtering, we apply this linear state space model to the data to maximize estimates for the posterior distribution of $\theta_{t}$.

When convergence is achieved for the likelihood and coefficient estimation, multiple imputations are drawn from the last posterior distribution. These imputations are used to conduct the DF test. While conventionally the distribution of the test statistic would be used to pool results across multiple imputations using Rubin’s Rules (Little & Rubin, 2019), due to the lack of a closed form distribution for the Dickey–Fuller test statistic under the null, we pool test results across imputations by calculating the median test statistic. This method has been employed in other contexts and demonstrated to perform well when traditional methods of pooling results are unavailable, and is applicable without having to generate a computationally intensive number of multiple imputations (Bolt et al., 2022; Eekhout et al., 2017; van de Wiel et al., 2009). The list below provides a general framework for the procedure of the SSMimpute method (Cai et al., 2022) for unit root testing:

- Initialization: Generate initial imputations for missing values ${\hat{Y}}_{t}^{\left(0\right)}$ using Kalman filtering, and substitute imputations into the corresponding missing lagged values to eliminate missingness in the regressors.
- Maximization: In the *j*th iteration, apply the state space model to outcome with missing values $Y_{t}$ and the explanatory imputed lagged ${\hat{Y}}_{t-q}^{\left(j-1\right)},\ldots ,{\hat{Y}}_{t-1}^{\left(j-1\right)}$ to obtain maximum likelihood estimates for the coefficients of regressors.
- Substitution: Calculate imputations of $Y_{t}^{\left(j\right)}$ from the updated posterior distribution and substitute these imputations into the corresponding lagged variables ${\hat{Y}}_{t-q}^{\left(j-1\right)},\ldots ,{\hat{Y}}_{t-1}^{\left(j-1\right)}$.
- Check convergence: Repeat steps 2 and 3 until convergence is reached for likelihood and coefficient estimation.
- Multiple imputation: Once convergence is achieved in iteration *J*, obtain *M* random draws of coefficient estimates from the posterior distribution of $\theta_{t}$ reached in iteration *J*. From each set of coefficient estimates, calculate imputations ${\hat{Y}}_{t}^{\left(J,m\right)}$.
- Unit root testing: Apply DF test to each imputation ${\hat{Y}}_{t}^{\left(J,m\right)}$ for m=1,…,M and obtain the resulting test statistic. Pool across imputation results by calculating the median test statistic and its corresponding *p*-value.

The SSMimpute approach for unit root testing relaxes assumptions by not relying on a single lag order. Although this makes it slightly more computationally intensive, by not calculating multiple imputations within each iteration, it eases the computational burden without compromising on estimate accuracy (Cai et al., 2022). Additionally, Cai et al. (2022) demonstrated the method has low bias across a range of data generation scenarios. To determine the appropriate number of lags, we recommend fitting the model for a range of lag orders, and selecting the number of lags corresponding to the model with the lowest Akaike information criterion.

Full properties of the four proposed methods for testing unit root non-stationarity with missing data can be seen in Table 1.

**Table 1.** Properties of four proposed methods for unit root testing with missing data

	MLEEM	MLEN	MLENS	SSMimpute
Assumptions	Errors are independently and identically normally distributed; Lag order of one	Errors are independently and identically normally distributed; Lag order of one	Errors are independently and identically normally distributed; Lag order of one	Errors are normally distributed; Finite lag order
Advantages	Computational efficiency; Able to incorporate *δ* for sensitivity analyses	Computational efficiency; Very low type I error	Computational efficiency	Flexible modelling assumptions; Able to incorporate *δ* for sensitivity analyses
Limitations	Strict assumptions	Strict assumptions; Relatively low power	Strict assumptions	Relatively computationally intensive

*Note*. MLEEM = MLE with expectation maximization; MLEN = MLE with numeric optimization; MLENS = MLE with numeric optimization, scaled; SSMimpute = State space model with multiple imputation.

## 4 MNAR sensitivity analyses

Data are classified as missing not at random, or MNAR when the probability of not observing a time point is dependent on unobserved information. This is the most severe missing data classification. However, MNAR cannot be tested for due to its nature, as the very information needed to evaluate the assumption is missing. In the case of a univariate time series, we assume that data MNAR would signify that the probability of missing a time point is dependent on the value of that individual time point. In the psychiatric mobile health context, this missingness seems plausible, with perhaps participants being less likely to report a negative mood when in a depressive state. Because we are unable to observe or test for the presence or influence of missing not at random, sensitivity analyses are commonly used to assess the impact of MNAR data (Little & Rubin, 2019). Here, we consider sensitivity analyses which incorporate a range of *δ* values to represent the hypothesized difference between observed and unobserved time points. For example, if one hypothesized that missing observations were on average 3 units higher in scale than an observed data because no response is believed to indicate more severe depression, we would let δ=3. In practice, a range of reasonable *δ* values are selected to test the implications of data missing not at random across varying hypotheses of how the MNAR mechanism operates. We propose methods to conduct such a sensitivity analysis for unit root testing for the maximum likelihood estimation with expectation maximization and state space model with multiple imputation methods.

The MLEEM method’s sensitivity analysis within each iteration, recursively across time *t* adds a term to each imputation for missing value $y_{t}$ such that ${\hat{y}}_{t}^{\left(j\right)}={\hat{\rho }}_{\left(j-1\right)}{\hat{y}}_{t-1}^{\left(j\right)}\pm \delta_{t}$. Thus $\delta_{t}$ represents the hypothesized missing mechanism, or specifically the expected difference in $y_{t}$ given $r_{t}=1$ and $y_{t}$ given $r_{t}=0$. As our eventual goal is testing for unit root, we wish avoid creating any major jumps in the time series when imputing, to not disrupt autocorrelation estimates. To do so, we assume that for a missing gap from time points $y_{u}$ to $y_{v}$, the time series will add $\delta_{t}$ to imputations for the first half of time points $t=(u,u+1,\ldots ,u+\lfloor \frac{v-u}{2}\rfloor )$, and subtract $\delta_{t}$ for time points $t=(u+\lfloor \frac{v-u}{2}\rfloor +1,u+\lfloor \frac{v-u}{2}\rfloor +2,\ldots ,v)$. This will essentially create a rise and fall peak effect across each missing gap.

We focus on the context where $\delta_{t}$ is constant across *t*, however we additionally consider the case from Example 15.4 in Little and Rubin (2019) where it is hypothesized that all missing values fall between (λ,∞). Note that in this scenario, for a sensitivity analysis *λ* would be chosen as a range of plausible values which would subsequently inform $\delta_{t}$. Under this hypothesis, conditioning on the normality assumption we can further inform our *δ*. We let *ϕ* and *Φ* represent the standard normal density and cumulative distribution functions respectively, and obtain the following adjustment:


$$\delta_{t}^{\left(j\right)}=\frac{\sigma^{\left(j\right)}\phi \left(\frac{\lambda -{\hat{\rho }}_{\left(j-1\right)}{\hat{y}}_{t-1}^{\left(j\right)}}{\sigma^{\left(j\right)}}\right)}{1-\Phi \left(\frac{\lambda -{\hat{\rho }}_{\left(j-1\right)}{\hat{y}}_{t-1}^{\left(j\right)}}{\sigma^{\left(j\right)}}\right)}.$$

For the SSMimpute approach, following initialization, we incorporate *δ* within the E-step of each iteration of the algorithm. After the imputation of missing values in lagged regressors, we will add a *δ* informed term to ensure all imputations are shifted to represent the hypothesized MNAR effects. Thus if the state space model in iteration *j* imputes for lagged regressor $Y_{t-q}$ at time point *t* a value ${\tilde{y}}_{t-q}^{\left(j\right)}$, we calculate the final imputation in this iteration to be ${\hat{y}}_{t-q}^{\left(j\right)}={\tilde{y}}_{t-q}^{\left(j\right)}+m_{t-q}\delta_{t-q}$. Similarly to the MLEEM sensitivity analysis, we wish to create a rise and fall of imputed values, to avoid a large jump in the imputed time series. Thus for a missing gap in between time points *u* and *v*, we let $m_{t}=(t-u+1)$ for $t=(u,u+1,\ldots ,u+\lfloor \frac{v-u}{2}\rfloor )$, and $m_{t}=(v-u-t)$ for $t=(u+\lfloor \frac{v-u}{2}\rfloor +1,u+\lfloor \frac{v-u}{2}\rfloor +2,\ldots ,v)$. We will refer to this adjustment as peak *δ*. We additionally consider the case where a value missing has a stagnant effect, and let $m_{t}=1$ across all time points ( $\delta_{s}$). As by the time the algorithm converges the *δ*-adjustment should be incorporated into the posterior, we do not additionally impose the adjustment to the multiple imputations sampled from said posterior.

## 5 Recommendations for analysis of processes with a unit root

After testing for unit root non-stationarity and failing to reject the null hypothesis of unit root, there are two broader approaches to be taken to perform valid subsequent statistical analyses. The first involves decomposing the tested time series, and using the decomposed information for analysis. In the case of unit root, researchers commonly employ differencing, or subtracting each time point from the prior point (Dickey et al., 1986). This would result in a new time series $Y_{t}^{\prime }$ to be used in subsequent analysis where for t = 2,…,T, $y_{t}^{\prime }=y_{t}-y_{t-1}$. It is important to note that differencing does not guarantee stationarity, and in some cases it may be necessary to perform second or larger order differences to obtain a time series without unit root (Granger & Newbold, 2014). In general, after differencing it is recommended to again test for unit root to ensure that the new series is stationary. Additionally, differencing or any type of decomposition will affect the interpretability of subsequent results. For example with a first difference, all results must be interpreted with respect to changes in the differences between subsequent time points of the time series, rather than changes in the values of the original observations. Lastly in the case of missing data, differencing will inflate the missing rate, as for $Y_{t}^{\prime }$ to be observed both $Y_{t}$ and $Y_{t-1}$ must be observed.

The second approach for analysing data with a unit root is to employ methods which are suitable for non-stationary processes. One such statistical tool is a state space model, whose flexibility allows for the presence of unit roots (Aoki, 2013; Bauer & Wagner, 2012). While most state space models were developed assuming fully observed data, there is a growing body of literature that provides a framework for employing a state space model under missing data (Cai et al., 2022; Van Lint et al., 2005). Such models can be applied to the data of a single individual, or across participants (Linderman et al., 2019; Lodewyckx et al., 2011). While methods that allow for non-stationarity may be more computationally intensive and statistically complex, they provide a path for analysing data as it was observed, without requiring decomposition and loss in interpretability.

## 6 Simulation settings

To assess the performance of proposed methods against existing missing data approaches for univariate time series, we conduct a simulation study, divided in two parts. The first applies methods under the hypothesis that data are MCAR or MAR, and the later employs the proposed sensitivity analyses, under the assumption data are MNAR.

### 6.1 Main simulation

We conduct 2,000 simulations generating time series with T=500 and a single lagged order such that $y_{t}=\rho y_{t-1}+\epsilon_{t}$ and $\epsilon_{t}∼N(0,1)$. For each simulation, we consider ρ=0.5,0.9,0.95,1. Within each simulation for the four time series, we consider MCAR, MAR, MNAR missing mechanisms, with missing rates of 30%, 50%, and 70%. For MCAR data, we determine missingness by a random sample of *T* from a binomial distribution with fixed p=0.3,0.5,0.7. For MAR, we specify p=c×t, with *c* set such that on average the desired missing rate is achieved, and the probability of missing increases as *t* increases. Lastly, to simulate MNAR, we first consider an extreme deterministic scenario, where values of $y_{t}$ above the 30th, 50th, or 70th quantile are all classified as missing (MNAR-D). We additionally for this simulation consider data such that probability of missing, *p* is calculated as an interaction between $t^{2}$ and $y_{t}$ (MNAR-T). Specifically, we calculate $p=e^{ct^{2}y_{t}}/(1+e^{ct^{2}y_{t}})$, with *c* defined such that on average the desired missing rate is achieved.

For each resulting time series, we apply mean imputation (M), LOCF, linear and spline interpolation (IntL, IntS), Kalman smoothing imputation (K) from the imputeTS package in R (Moritz & Bartz-Beielstein, 2017), and complete case analysis (CC) including only observed time points. For each of these single imputations and complete case analysis we apply the DF test. We additionally include multiple imputation with chained equations (MICE). We consider two variations of covariates: one using only $X_{t}=(Y_{t-1},t)$, and another with $X_{t}=(Y_{t-1},Y_{t-2},Y_{t+1},Y_{t+2},t)$. For both, we set m=5, perform analysis using the mice R package (van Buuren & Groothuis-Oudshoorn, 2011), and obtain a pooled result for the Dickey–Fuller test by taking the median test statistic. We compare these methods with results from MLEN, MLENS, MLEEM, and SSMimpute with m=5, and a single lag order. For MLEEM, we defined convergence as less that 0.01 changes in the added absolute differences for $\hat{\rho }$ and $\hat{\sigma }$ between subsequent iterations. For SSMimpute, convergence was reached when both the sum of the absolute value of changes in estimates and the absolute difference in log likelihood were less than 0.01. For initial values for SSMimpute, we conservatively chose intercept $\beta_{0}=0$, and $\beta_{1}=\rho =1$, and initial values of $W_{t}=\text{diag}(1,000,1,000)$.

In addition to extracting the test statistic and *p*-value for each simulation, we calculate the estimated autocorrelation, $\hat{\rho }$. To evaluate the methods, we examine autocorrelation, test statistic, and *p*-value distributions across *ρ*, and the power across different values of ρ<1 and type 1 error when ρ=1.

### 6.2 Sensitivity analysis simulation

To evaluate proposed sensitivity analyses methods, we ran 1,000 simulations with T=500, for ρ=0.5,0.9,0.95,1. For the simulation we consider four different MNAR mechanisms. First, we consider MNAR-D and MNAR-T, as described above. We additionally consider, MNAR-P, which uses a probabilitic framework such that the time series observations were divided into three quantiles with 40%, 10%, and 0% missing rates, in descending order of quantile. The fourth, MNAR-H, a hybrid between probabilistic and deterministic missing, added an additional quantile to MNAR-P in which all data was missing. As in the main simulation, we consider 30%, 50%, and 70% missing rates, and set quantile cutoffs accordingly. For MLEEM, we consider $\delta_{t}=0.05,0.1,0.2,0.3$ with a fixed *δ* across time. For SSMimpute, we consider a peak $\delta_{t}=0.05,0.1,0.2,0.3$. We do not include the stagnant *δ* adjustment for SSMimpute due to convergence issues.

## 7 Simulation results

The percent of simulations which rejected the null of unit root in the main simulation are shown in Table 2 by missing rate, method, missing mechanism (MCAR, MAR, MNAR-D), and *ρ*. For ρ=1, the value represents type I error, while for ρ<1, the value is equivalent to power. The entry with rate of 0 for each *ρ* under CC represents the performance of the DF test with no missing data. We omit the case where ρ=0.5, as here all methods have power greater than 0.95. Additionally, mean imputation and both versions of MICE are omitted, but across all simulations these methods had a type I error greater than 0.7, demonstrating their ineffectiveness for unit root testing. The MLEN method is indeed very conservative, with type I error of 0 for MCAR and MAR data, and low power. When data are MCAR or MAR, MLEEM, MLENS, and SSMimpute have power over 79% for a *ρ* of 0.95. These methods additionally demonstrate type I error less that 0.05 with a missing rate of 30% when data are MCAR or MAR. Complete case analysis also has comparable type I error, but power suffers with higher rates of missing data, as expected. When data is MAR with 70% missing, the complete case power is merely 32%, compared to 96% and 85% for MLENS and SSMimpute, respectively. We observe that LOCF exhibits both inflated type I error and reduced power, while linear and spline (excluded from table) interpolation and Kalman smoothing imputation both bias towards non-stationarity and have low power. For a deterministic missing not at random, or MNAR-D, time series, all methods perform poorly as expected, given they are misspecified. Regardless of method, even with only 30% missing not at random, type I error is greater than 8%, meaning an increase chance of rejecting the null of non-stationarity when the time series is truly non-stationary. With SSMimpute, this effect is especially severe, with a type I error of 30%. For time series with probability of missing as an interaction of value and time, MNAR-T, and moderate missing rates, methods no longer exhibit an inflated type I error, but power does suffer, particularly for complete case analysis.

**Table 2.** Simulated power (when ρ<1) and type I error (when ρ=1) by missing mechanism, *ρ*, missing rate, and method

	*ρ*	Rate	CC	MLEEM	MLEN	MLENS	SSM	LOCF	IntL	K
MCAR	1	0	0.05							
		0.3	0.05	0.05	0	0.01	0.05	0.23	0.01	0.05
		0.5	0.06	0.05	0	0.01	0.06	0.21	0.01	0.06
		0.7	0.08	0.05	0	0.02	0.08	0.21	0.01	0.08
	0.95	0	0.83							
		0.3	0.81	0.82	0.65	0.92	0.83	0.81	0.36	0.38
		0.5	0.78	0.81	0.42	0.97	0.83	0.77	0.08	0.10
		0.7	0.72	0.80	0.04	0.98	0.85	0.67	0	0.08
	0.90	0	1							
		0.3	1	1	1	1	1	1	0.99	0.99
		0.5	1	1	0.99	1	1	1	0.80	0.84
		0.7	1	1	0.63	1	1	1	0.10	0.26
MAR	1	0	0.05							
		0.3	0.05	0.05	0	0.01	0.05	0.17	0.02	0.02
		0.5	0.05	0.06	0	0.05	0.07	0.13	0.01	0.02
		0.7	0.04	0.11	0	0.10	0.33	0.08	0.05	0.07
	0.95	0	0.83							
		0.3	0.83	0.82	0.13	0.95	0.83	0.79	0.36	0.39
		0.5	0.72	0.82	0.76	1	0.84	0.70	0.12	0.2
		0.7	0.32	0.79	0.76	0.96	0.85	0.70	0.24	0.3
	0.90	0	1							
		0.3	1	1	1	1	1	1	0.99	0.99
		0.5	1	1	1	1	1	1	0.84	0.90
		0.7	0.83	1	0.7	1	1	0.99	0.81	0.87
MNAR-D	1	0	0.05							
		0.3	0.12	0.13	0.08	0.17	0.30	0.17	0.10	0.12
		0.5	0.16	0.22	0.06	0.20	0.54	0.20	0.19	0.19
		0.7	0.18	0.35	0.03	0.19	0.82	0.39	0.39	0.31
	0.95	0	0.83							
		0.3	0.94	0.96	0.88	0.96	0.98	0.94	0.92	0.93
		0.5	0.91	0.96	0.44	0.81	0.99	0.97	0.96	0.95
		0.7	0.77	0.91	0.03	0.33	0.99	0.98	0.97	0.78
	0.90	0	1							
		0.3	1	1	0.99	1	1	1	1	1
		0.5	1	1	0.60	0.96	1	1	1	1
		0.7	0.98	1	0.01	0.40	1	1	1	0.99
MNAR-T	1	0	0.05							
		0.3	0.05	0.05	0	0.01	0.06	0.16	0.02	0.02
		0.5	0.06	0.07	0	0.05	0.13	0.13	0.03	0.04
		0.7	0.05	0.19	0	0.10	0.55	0.13	0.13	0.16
	0.95	0	0.83							
		0.3	0.80	0.83	0.84	0.97	0.84	0.80	0.39	0.44
		0.5	0.55	0.81	0.60	0.97	0.82	0.71	0.35	0.40
		0.7	0.18	0.80	0.08	0.87	0.88	0.72	0.47	0.51
	0.90	0	1							
		0.3	1	1	1	1	1	1	0.99	1
		0.5	0.99	1	0.99	1	1	0.99	0.95	0.97
		0.7	0.62	1	0.48	1	1	0.99	0.93	0.95

*Note*. CC = complete case analysis; SSM = SSMimpute; LOCF = last observation carry forward; IntL = linear interpolation, and K = Kalman smoothing imputation.

**Figure 1.**
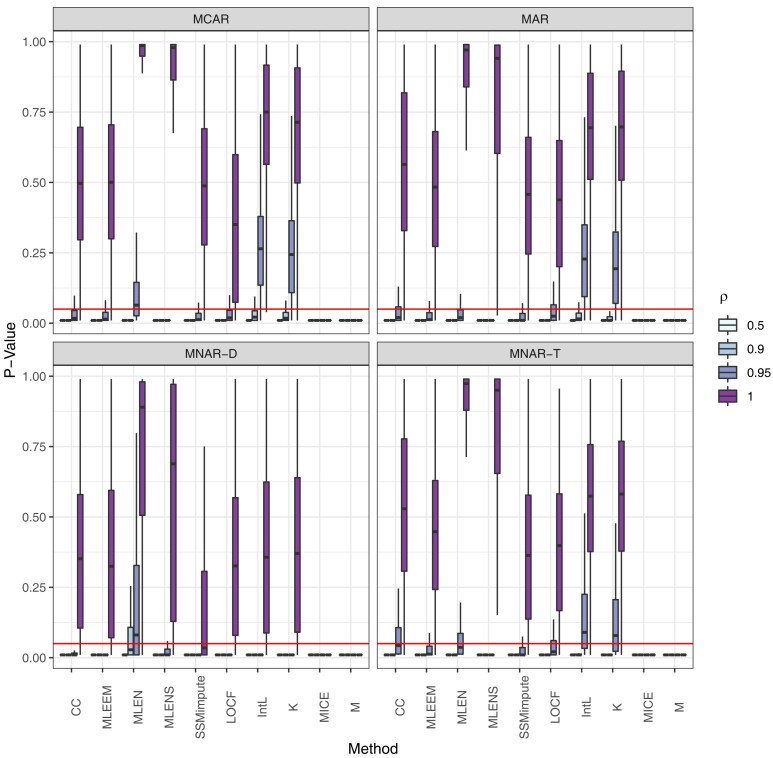
*P*-value box plots by missing mechanism, *ρ*, and method for time series with 50% missing. The horizontal line indicates cut-off values of α=0.05. CC = complete case analysis; LOCF= last observation carry forward; IntL = linear interpolation; K = Kalman smoothing imputation; M = mean imputation.

**Figure 2.**
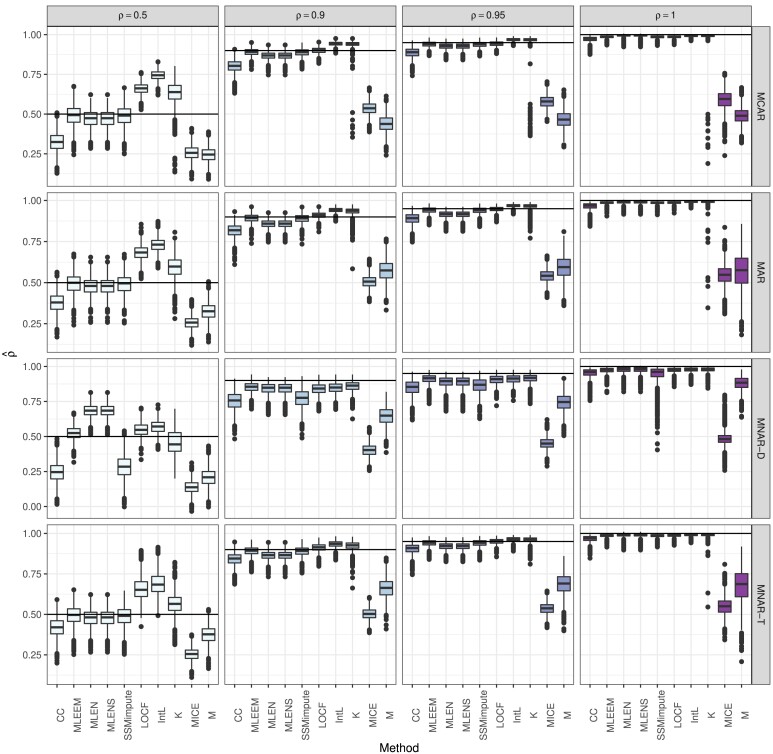
Autocorrelation estimates for time series with 50% missing by missing mechanism, true *ρ*, and method. The horizontal lines indicate the true value of ρ.

The box-plots of *p*-values by method and missing mechanism with 50% missing in Figure 1 further demonstrate simulation results. High power is seen by box-plots for ρ<1 mostly below the α=0.05 cutoff, and low type I error is visualized by little of the ρ=1 distribution of *p*-values below the α=0.05 line. For clarity, we omit on the case of MICE using five covariates ( $Y_{t-1},Y_{t-2},Y_{t+1},Y_{t+2},t$) in graphs but note that its performance had only a slightly edge over MICE with two covariates ( $Y_{t-1},t$), and produced an unacceptably high level of type I error. Across all methods, the MNAR-T mechanism does not suffer from the high type I error seen for MNAR-D, but does have lower power compared to MCAR and MAR. In Figure 2, the $\hat{\rho }$ estimates by method are plotted, demonstrating the biases by method and missing mechanism. The four proposed methods, SSMimpute, MLEEM, MLEN, and MLENS all have low bias when data are MCAR or MAR. As seen with *p*-values, mean and MICE imputation underestimate *ρ*. LOCF, linear interpolation, and Kalman smoothing imputation bias results towards non-stationarity, as is seen by their estimates above the true value of *ρ*, when ρ<1. As expected, complete case analysis underestimates *ρ* when ρ<1, and is accurate when ρ=1. In online supplementary material, Web Appendix A, we include graphs for the *p*-values, $\hat{\rho }$ estimates, and test statistics with 30%, 50%, and 70% missing across methods, *ρ*, and missing mechanism.

**Figure 3.**
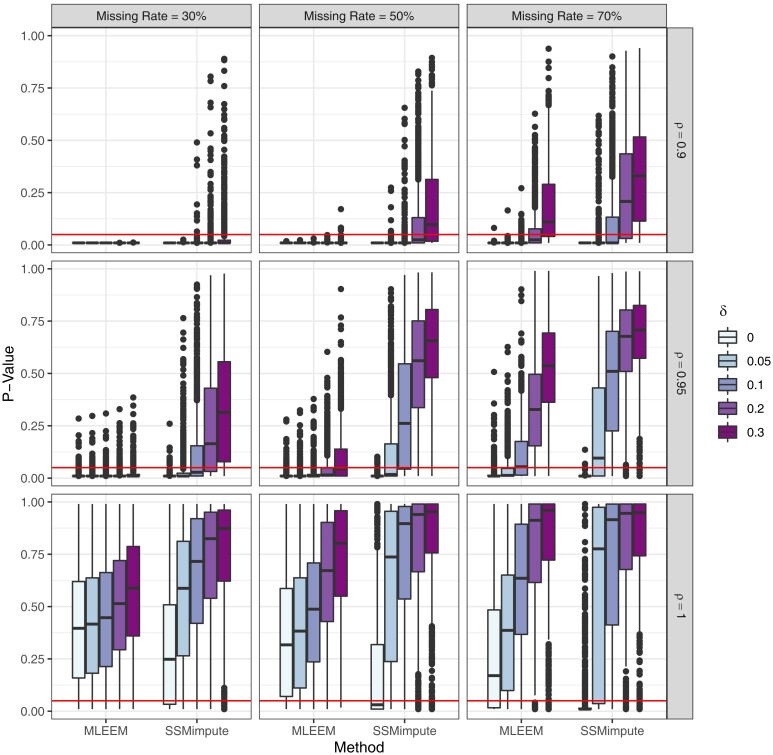
*P*-value box plots for sensitivity analysis by missing rate, true *ρ*, method, and *δ* adjustment. The horizontal line indicates cut-off values of α=0.05.

In the sensitivity analysis simulation, we see that across different types of MNAR data the proposed *δ* adjustments are able to shift results from the DF test. Specifically, we demonstrate that with positive *δ* values, we are able to correct the inflated type I error exhibited by MNAR-D data in the general simulation. In Figure 3, *p*-values for the SSMimpute and MLEEM methods are visualized across a range of *δ* values for MNAR-D time series. Increases in *δ* generate a increase in *p*-value for both methods, representing a reduced likelihood of rejecting the null hypothesis and thus a shift toward non-stationarity. Simulation results additionally demonstrate that the SSMimpute method appears more sensitive to changes in *δ* compared to MLEEM. This was expected, as it is a more flexible model and thus more able to adapt to reflect changes in the imputation structure. The results for hybrid and probibalistic missing not at random are similar to those seen with deterministic missing not at random. For MNAR-T, we did not see the same pattern of inflated type I error in prior to sensitivity analyses, and the proposed *δ* adjustments tested fail to incorporate the impact of *t* on the probability of missing. Despite these limitations, the increasing *δ* adjustments do provide an increasing range of *p*-value and autocorrelation estimates. This is especially pronounced when applied to time series with higher rates of missing data. Full visualizations across tested MNAR mechanisms are shown in online supplementary material, Web Appendix B, with autocorrelation and *p*-value graphs by missing mechanism, missing rate, and *ρ*.

Overall when the time series is MCAR or MAR, the simulation demonstrates the high power and acceptable type I error of proposed methods, and the limitations of existing methods when testing for unit root non-stationarity. We additionally observe inflated type I error across all methods when data are MNAR. The impact of time points missing not at random can be assessed using a sensitivity analysis with the MLEEM or SSMimpute methods by specifying a range of *δ* adjustments.

## 8 Application to mobile health data

We applied the proposed methods for unit root testing in univariate time series under missing data to mobile health data collected from the BLS. BLS is an ongoing longitudinal cohort study of 74 participants with Bipolar I or II disorder, schizophrenia, or schizoaffective disorder recruited from the Psychotic Disorders Division at McLean Hospital in Belmont, MA. The Beiwe mobile application developed by Jukka-Pekka Onnela’s lab (Barnett & Onnela, 2020; Huang & Onnela, 2019; Onnela et al., 2021) is employed to passively collect data on physical activity, GPS locations, and telecommunications of texts and calls. The application additionally is configured to prompt users to respond daily to a 5-min survey at 5p.m. on their moods, sleep, social activity, and psychotic symptoms. To apply proposed methods, we focus on negative and positive mood scores which are built as aggregates of survey responses to questions. Negative mood score is generated from questions relating to fear, anxiety, embarrassment, hostility, stress, upset, irritation, and loneliness, ranging from 0 to 27 (Cai et al., 2022). Positive mood score is from questions relating to stress management, determination, and being alert, energetic, happy, inspired, and outgoing, with scores ranging from 0 to 28. Across participants, missing rates in mood scores vary from less than 10% to over 90%. We applied new methods SSMimpute, MLEEM, MLEN, MLENS, and compare them to existing methods of complete case analysis, LOCF, linear and spline interpolation, Kalman smoothing imputation, mean imputation, and MICE for unit root testing. We additionally considered MNAR senstivity analyses for the SSMimpute and MLEEM methods. As the mood score errors are not normally distributed, we found MLE methods conditioning on normality were unfit, and tended to over-report non-stationarity and estimate *ρ* close to 1. To evaluate the performance of other methods, we highlight the time series of four participants.

**Figure 4.**
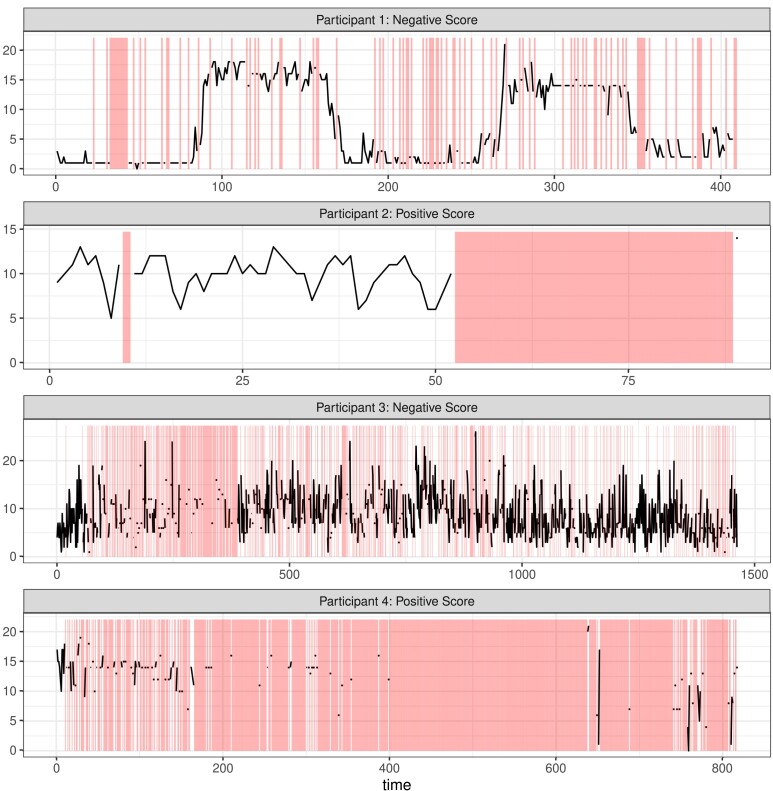
Time series plots for four participants included in BLS study. Missing observations are marked by shading.

We first focus on the negative mood score for a participant with bipolar disorder followed for 708 days, with a 22.6% missing rate in negative mood. We will concentrate on the first 407 days of follow up, prior to a significant life change for the patient which created a change point in the observed data. As shown in Figure 4, the negative mood time series of participant 1 appears non-stationary with potential seasonality. All methods but mean and both versions of MICE imputation had a *p*-value greater than 0.05, and thus failed to reject the null hypothesis of unit root. These results concord with those found in the simulation, where MICE and mean imputation strongly bias test results towards stationarity regardless of the true *ρ*. For reference, all but mean imputation and MICE estimated the autocorrelation to be greater than 0.95. As expected, a sensitivity analysis for the SSMimpute and MLEEM methods with *δ* values ranging from 0 to 1 had no impact on failing to reject the null of non-stationarity.

We additionally consider the positive mood score of a participant with bipolar disorder with 89 days of follow up and a 41.6% missing rate in positive mood observations. In Figure 4, we see for the positive score of participant 2 almost all missing time points are consecutive, with only one observation following the missing block. Realistically, viewing this time series we would likely decide to omit the final observation and only use data collected prior to the missing gap. If we were to do so, all methods would reject the null and find the time series to be stationary, with autocorrelation estimates ranging from 0.2 to 0.5. Results do not change across the MNAR sensitivity analysis.

However, if we blindly apply methods to the entire follow up period, we obtain different results. Spline interpolation, Kalman smoothing imputation, MLEN, and MLENS now reject the null of unit root. This reflects results seen in the simulation, where spline interpolation and Kalman smoothing imputation appear to bias towards non-stationarity, reflected by an inflation in the type I error. Comparing autocorrelation estimates, those of linear and spline interpolation and Kalman smoothing imputation are larger than 0.6, while for complete case analysis, SSMimpute, and LOCF they are less that 0.4. When considering values of *δ* from 0 to 1 for a MNAR senstivity analysis, MLEEM with a δ≥0.5 fails to reject the null hypothesis. SSMimpute shows increased estimates of autocorrelation, but across all considered *δ* concludes that the time series is stationary. This example of a short time series with high rates of missing data demonstrates how each time series should be considered individually and the importance of reasonably choosing the most appropriate method to handle missing data. In this case, the most reasonable approach is not to use a sophisticated imputation method but rather to ignore the second half of the follow up period where only one time point was observed.

Next, we include the negative mood score of a participant with schizophrenia and 1,461 days of follow up with 34.6% missing. In Figure 4, the time series visually appears stationary, with intermittent missingness. Indeed, all methods reject the null hypothesis of unit root and find the autocorrelation to be less than one. This example demonstrates that with sufficiently low value of *ρ*, a relatively low missing rate, and a long follow up, the power is high enough that the chosen method to handle missingness has no impact on unit-root testing. Even in sensitivity testing, we found little changes in autocorrelation estimates and no changes by Dickey–Fuller test results across *δ* values.

Lastly, we have the positive mood score of a participant with bipolar disorder and a long follow up yet sparse information. There is a follow up of 861 days of which 84.5% are missing. In Figure 4, it is hard to discern any pattern regarding stationarity with so much missing data. When we apply all methods, spline interpolation, MLEN, and MLENS fail to reject the null of unit root, and all others reject the null. However, the meaning or validity of these results is questionable with so little actual data observed. It is difficult to discern the true autocorrelation of this time series, with estimates ranging from 0.1 to 0.99 across methods. This case demonstrates how much leverage is left to the method with a high rate of missingness, and an example where testing for unit root may not be feasible.

## 9 Discussion

The increase in not fully observed time-series data from contexts such as mHealth necessitates the adaptation of current time series analysis methods to a missing data setting. In particular, testing a univariate time series for stationarity is an important evaluation to inform appropriate subsequent analyses. However, there are few recommendations for testing unit root with missing data, and no consideration for missing mechanisms beyond missing completely at random. Here we propose adaptations to the DF test including an MLE approach for testing for unit root when the time series has lag order of one and normal and independent errors, and a state space model multiple imputation method to test for unit root for processes with longer lag orders. We additionally introduce sensitivity analyses to evaluate the impact of data MNAR on test results and autocorrelation estimation. In our simulation we find that both methods are effective under MCAR and MAR mechanisms, with improved power and reasonable type I error when missingness is moderate. We additionally show that our proposed sensitivity analyses are effective at generating a range of autocorrelation estimates and test results depending on the missing mechanism assumptions. By applying the existing univariate time series missing methods and our proposed methods to mood time series from the Bipolar Longitudinal Study, we demonstrate that our simulation results largely hold in observed data. We additionally see the limitation of the proposed MLE methods when errors are not normally distributed.

The proposed SSMimpute, MLEEM, and MLENS methods offer computational efficiency, with high power and acceptable type I error across MCAR and MAR time series with varying rates of missingness. SSMimpute and MLEEM additionally offer researchers the ability to evaluate the impact of hypothesized MNAR mechanisms on test results through sensitivity analyses. When noise is normally distributed and there is a single lag error, we recommend the MLENS method which demonstrates high power and low type I error when correctly specified. Should data be hypothesized to be missing not at random, the MLEEM method can be employed for sensitivity analyses. Should the time series follow a more complex generation process, the SSMimpute method is most appropriate, as it can handle lag order greater than one. These methods will allow for improved validity of future mHealth analyses by accurate unit root testing to inform model selection and appropriate assumptions.

One limitation of the simulation results presented is the simplicity in the data generation process. In real-data, it is unlikely that a time series is generated under a single lag. Despite this limitation, we have reason to believe the results found in the simulation will hold under more complex data generation patterns, as we find similar results between the application with real-data and the simulation in terms of methods and the biases they do or do not inflict on autocorrelation estimation. Notably, the SSMimpute method used with the augmented Dickey–Fuller test would be appropriate for a time series with a higher lag order, and maximum likelihood methods could likely be extended to this setting. We also perhaps simplistically apply the unit root test to the time series in the simulations and application without considering any prior decomposition. This is in part due to limitations in methods for time series decomposition under missing data. In the BLS application, we do attempt detrending each time series using polynomial regression (with order of three) on each imputation obtained from the state space model. We find that a unit-root is still identified for Participant 1, indicating that the unit root likely is not attributable to a trend. For the other participants results from the SSMimpute method were unchanged, all continued to reject the null of unit root. We note however, this approach for detrending would only be appropriate with a unit root test method which uses imputation and does not assume no trend when imputing. Additional research is needed to develop methods for detrending, adjusting for seasonality, and adjusting for covariate time series under missing data, all of which might be of interest prior to applying a test for unit root. We also note that more research is needed for unit-root testing with missing data under different distributional assumptions. All methods proposed here assume errors are normally distributed, and more generally the DF test is appropriate only for continuous time series variables. We further explore implications of normality violations in a supplementary simulation with t-distributed noise ( df=5), where we see a moderate reduction in power across existing and proposed methods. Full results from this simulation are shown in online supplementary material, Web Appendix C. We lastly note that Im et al. (2014) proposed a more powerful RALS-based unit root test for non-normally distributed errors, for which further work is needed to extend the test to a setting with missing data.

Additionally, the general limitations in power of the DF test, especially when autocorrelation is close to one or there are few observations (Harris, 1992) still apply to the proposed methods. We also find that with very high rates of missing data, all methods suffer from poor power, and in such cases it may be unfeasible to test validly for unit root. Lastly, the DF test tests only for unit root non-stationarity. This is also true of any proposed adaptations to address missing data. Further work is needed to extend the Kwiatkowski–Phillips–Schmidt–Shin (KPSS) test for trend stationarity, which employs an alternative hypothesis of unit root, to a context with missing data (Kwiatkowski et al., 1992). The SSMimpute algorithm might also be appropriate to generate imputations prior to applying the KPSS test, but more work is needed to validate this procedure. Other violations to stationarity such as change points and unstable variance could also be found in mHealth data and should also be tested for to inform subsequent analyses. Further research is needed to develop hypothesis tests for additional types of non-stationarity in the context of missing data. We lastly note that more research is needed to provide a framework for identifying the correct lag order under missing data, with considerations towards the different mechanisms of missingness.

## Supplementary Material

qlae010_Supplementary_Data

## Data Availability

Data used in this paper to illustrate the proposed methods are not shared due to privacy restrictions.
