# Supplementary material for: Testing unit root non-stationarity in the presence of missing data in univariate time series of mobile health studies
Source: J R Stat Soc Ser C Appl Stat. 2024 Feb 29;73(3):755–73. doi: 10.1093/jrsssc/qlae010 (PMC11175825; doi:10.1093/jrsssc/qlae010)
Supplement: qlae010_Supplementary_Data [file qlae010_supplementary_data.zip › Web Appendix A.pdf]

Web Appendix A: Simulation Results

P-Value Visualization

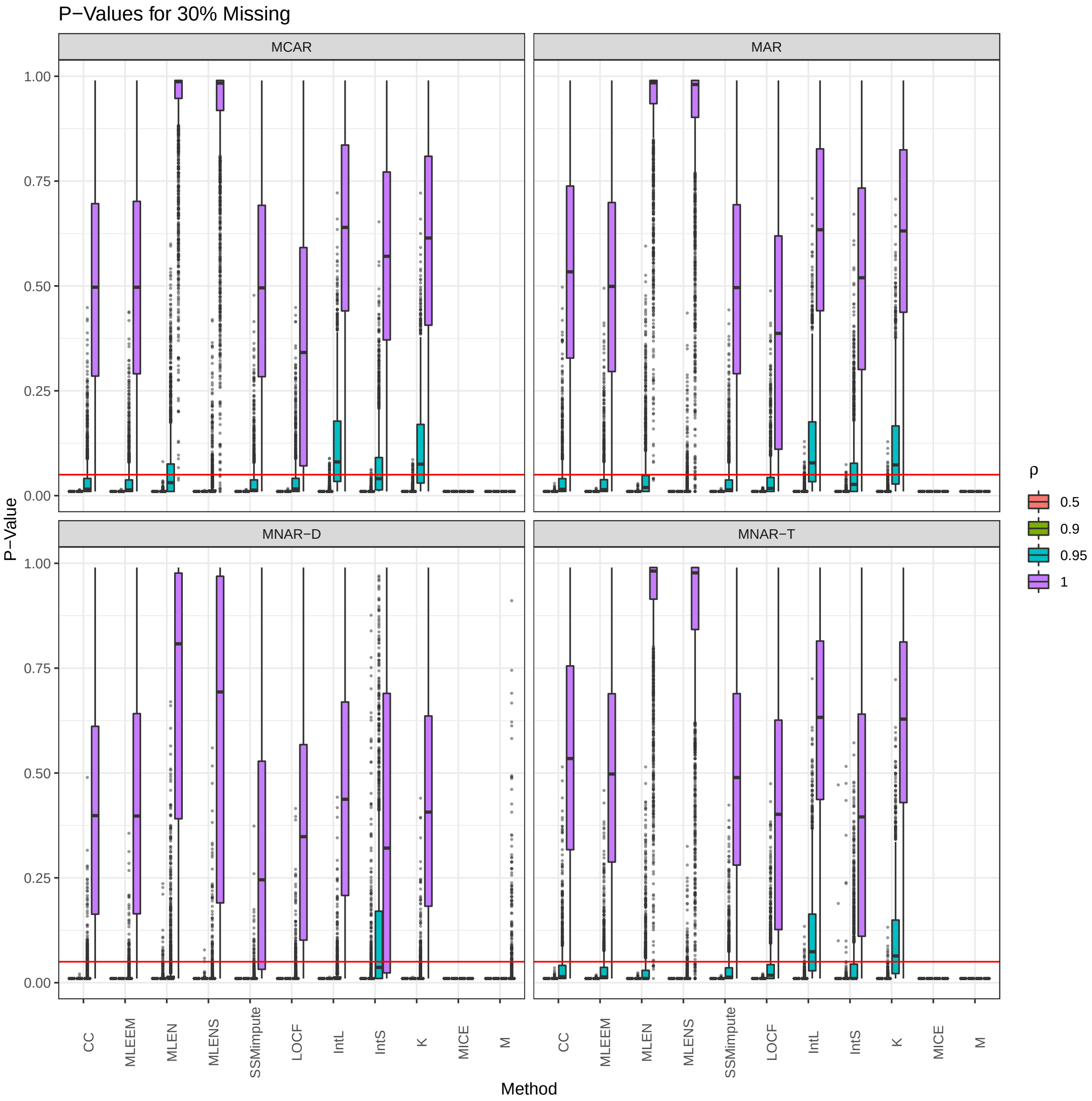

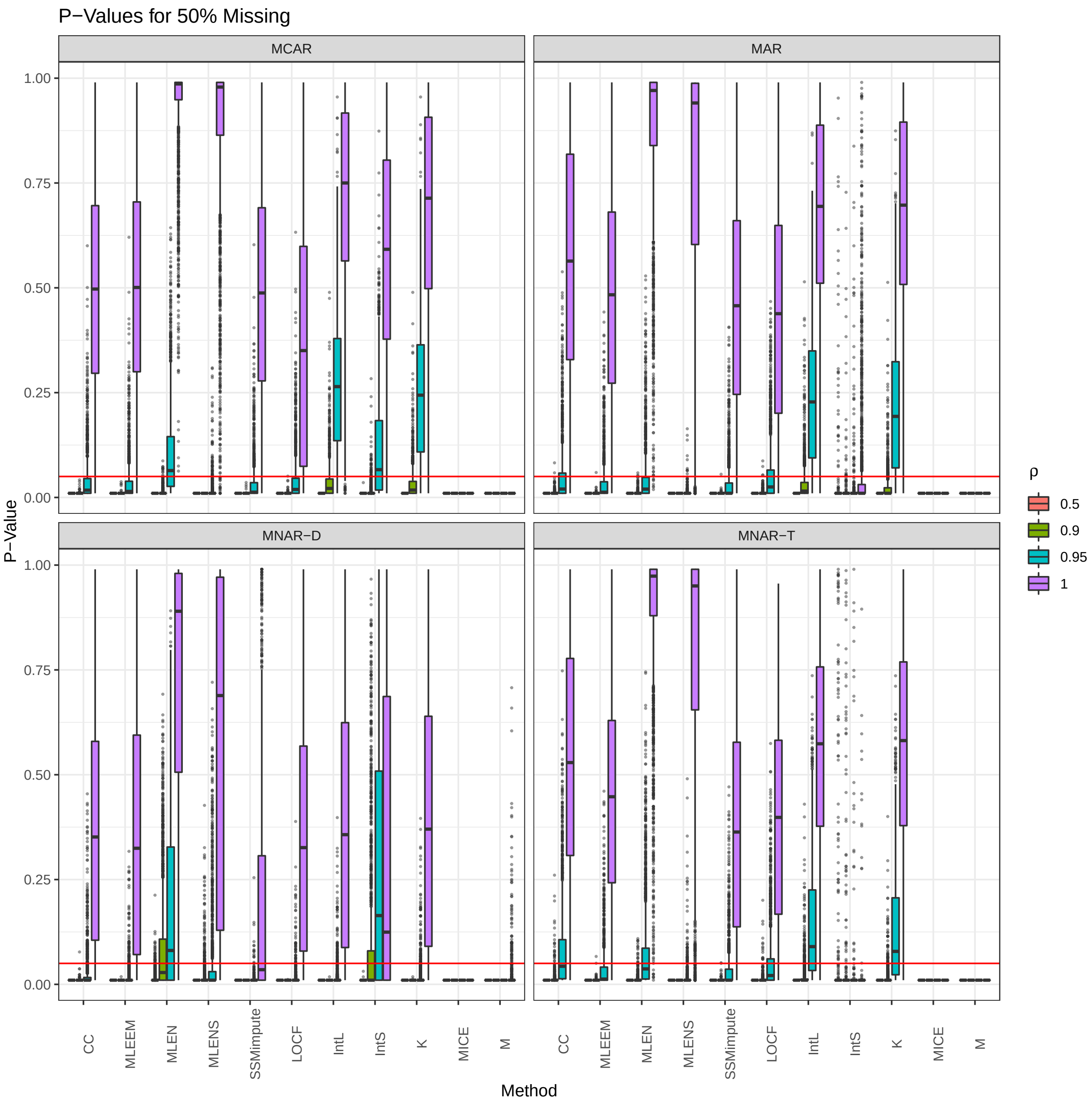
P−Values for 50% Missing
MCAR
MAR
MNAR−D
MNAR−T
P−Value
1.00
0.75
0.50
0.25
0.00
Method
CC
MLEEM
MLEN
MLENS
SSMimpute
LOCF
IntL
IntS
K
MICE
M
ρ
0.5
0.9
0.95
1

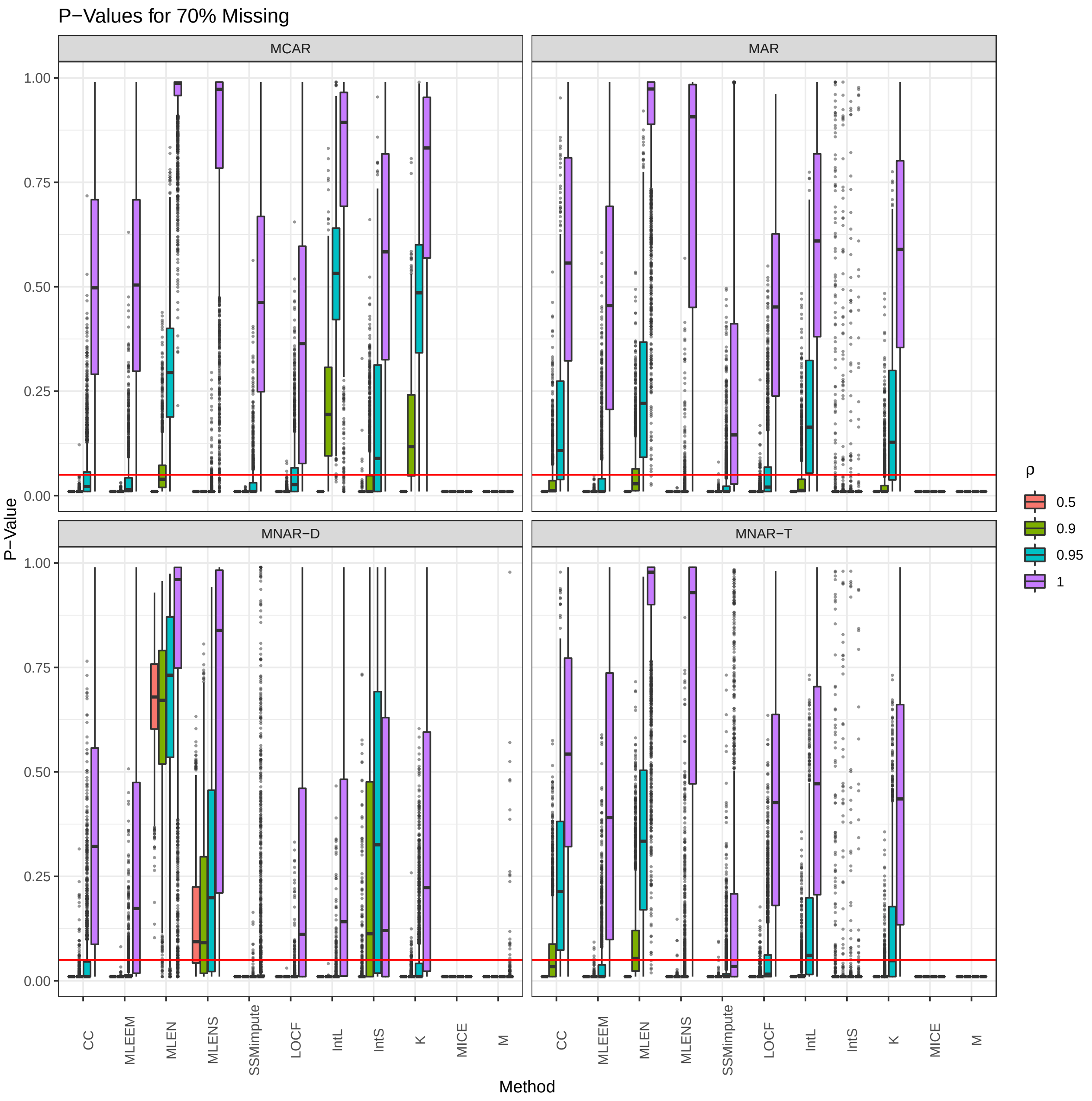
P−Values for 70% Missing
MCAR
MAR
MNAR−D
MNAR−T
P−Value
1.00
0.75
0.50
0.25
0.00
CC
MLEEM
MLEN
MLENS
SSMimpute
LOCF
IntL
IntS
K
MICE
M
Method
ρ
0.5
0.9
0.95
1

Test Statistic Visualization

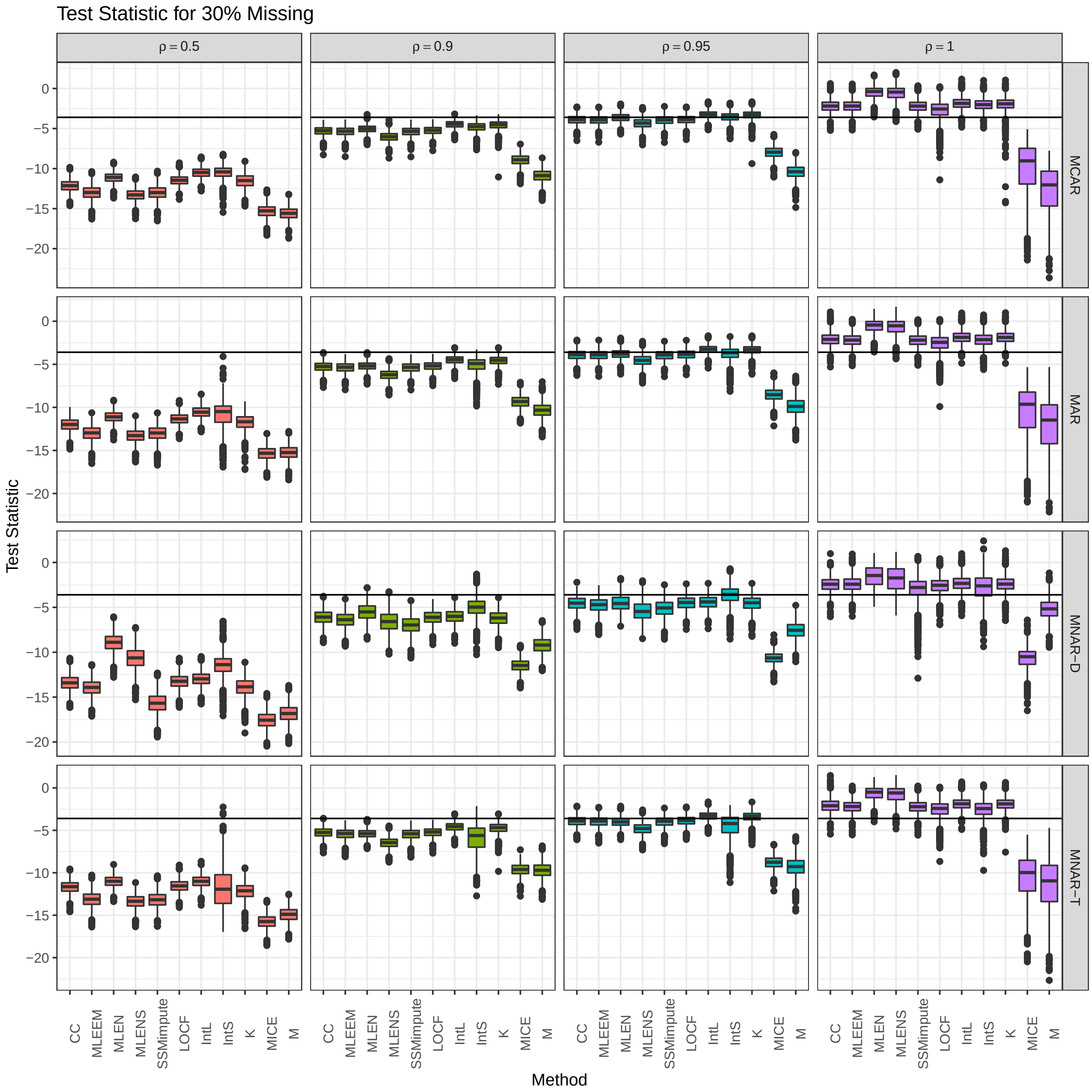

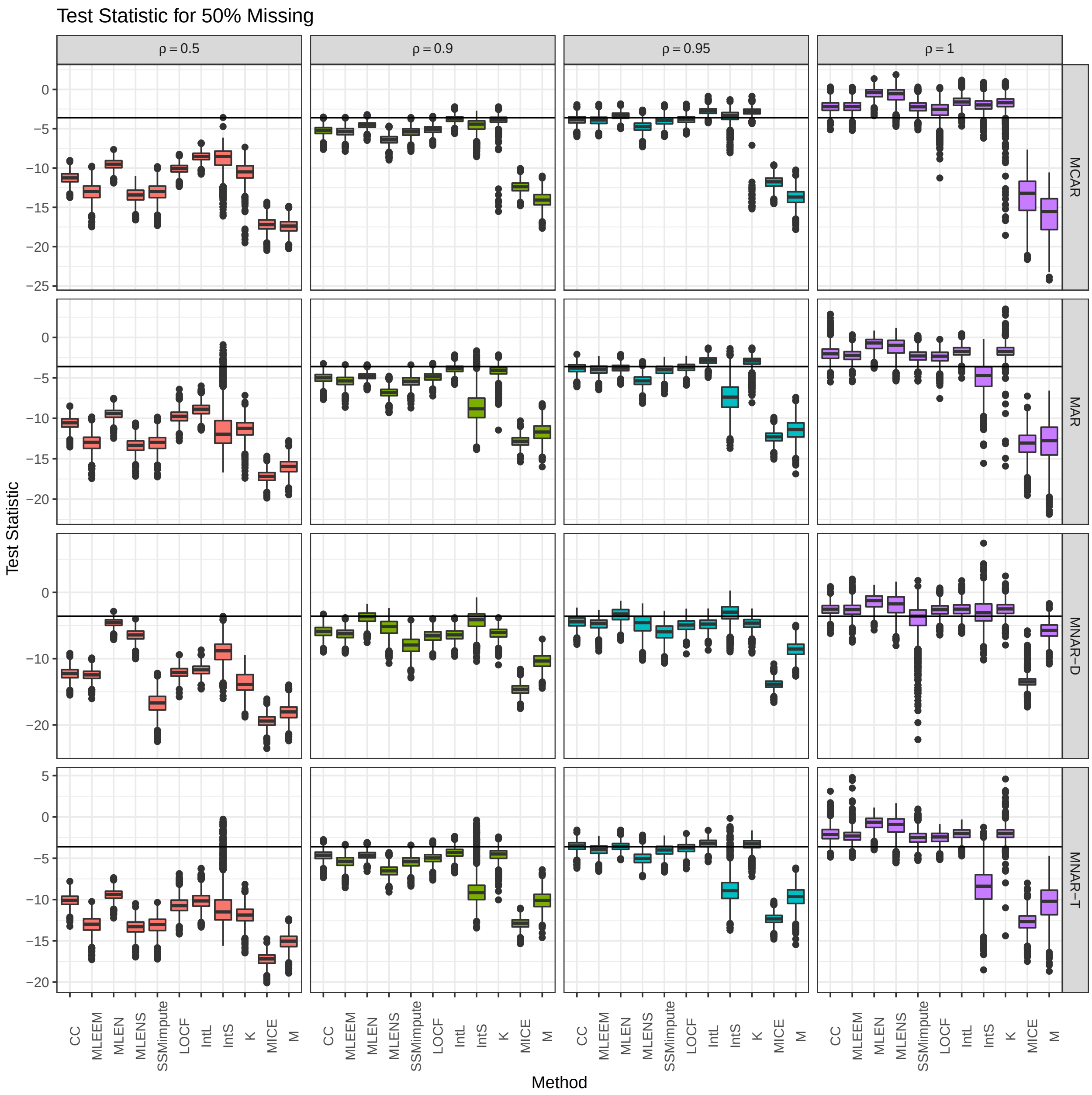

Test Statistic for 50% Missing
ρ = 0.5
ρ = 0.9
ρ = 0.95
ρ = 1
MCAR
MAR
MNAR−D
MNAR−T
Test Statistic
Method
CC
MLEEM
MLEN
MLENS
SSMimpute
LOCF
IntL
IntS
K
MICE
M

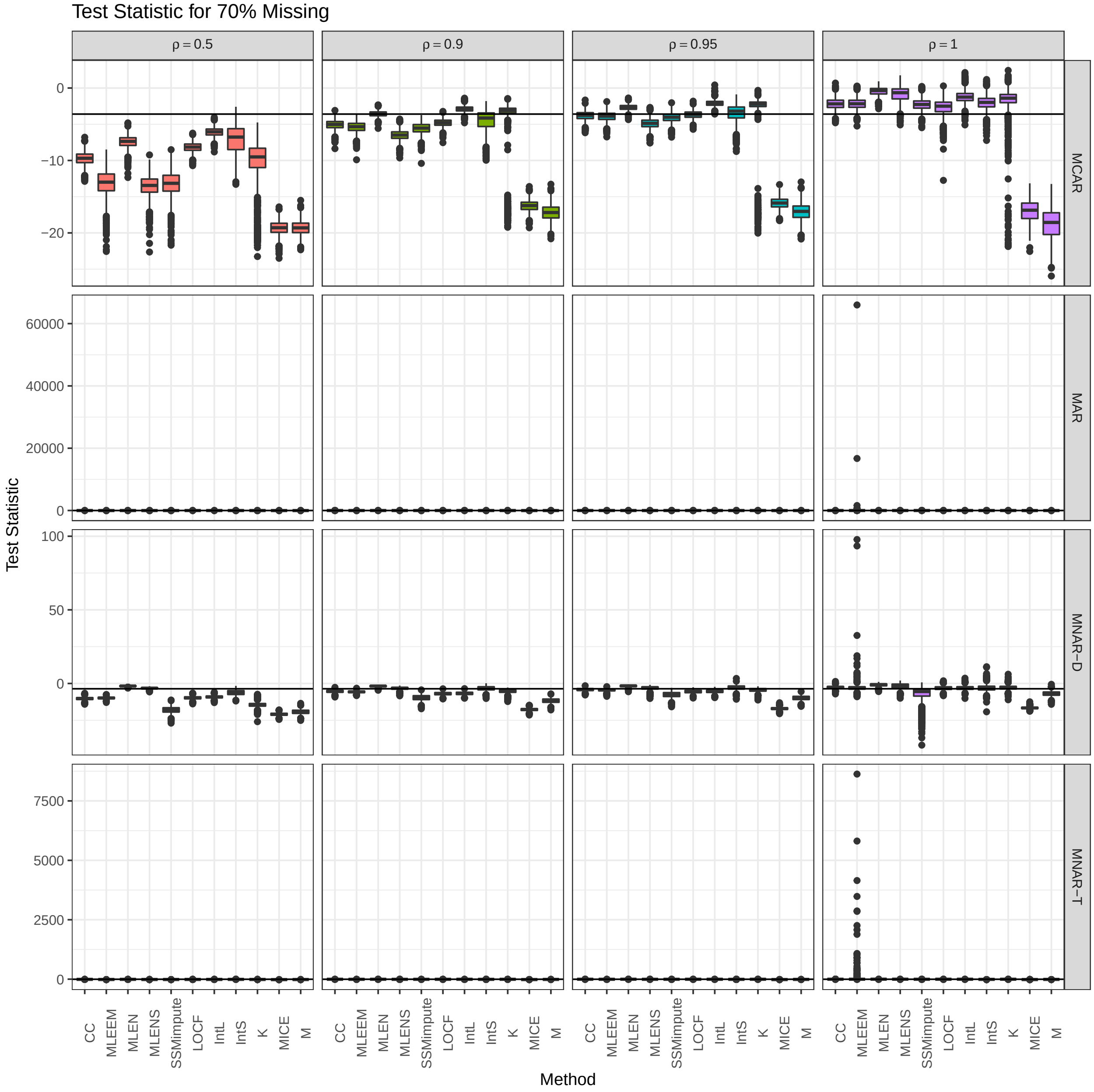

Test Statistic for 70% Missing
ρ = 0.5
ρ = 0.9
ρ = 0.95
ρ = 1
MCAR
MAR
MNAR−D
MNAR−T
Test Statistic
0
−10
−20
60000
40000
20000
0
100
50
0
7500
5000
2500
0
CC
MLEEM
MLEN
MLENS
SSMimpute
LOCF
IntL
IntS
K
MICE
M
Method

Autocorrelation Vizualization

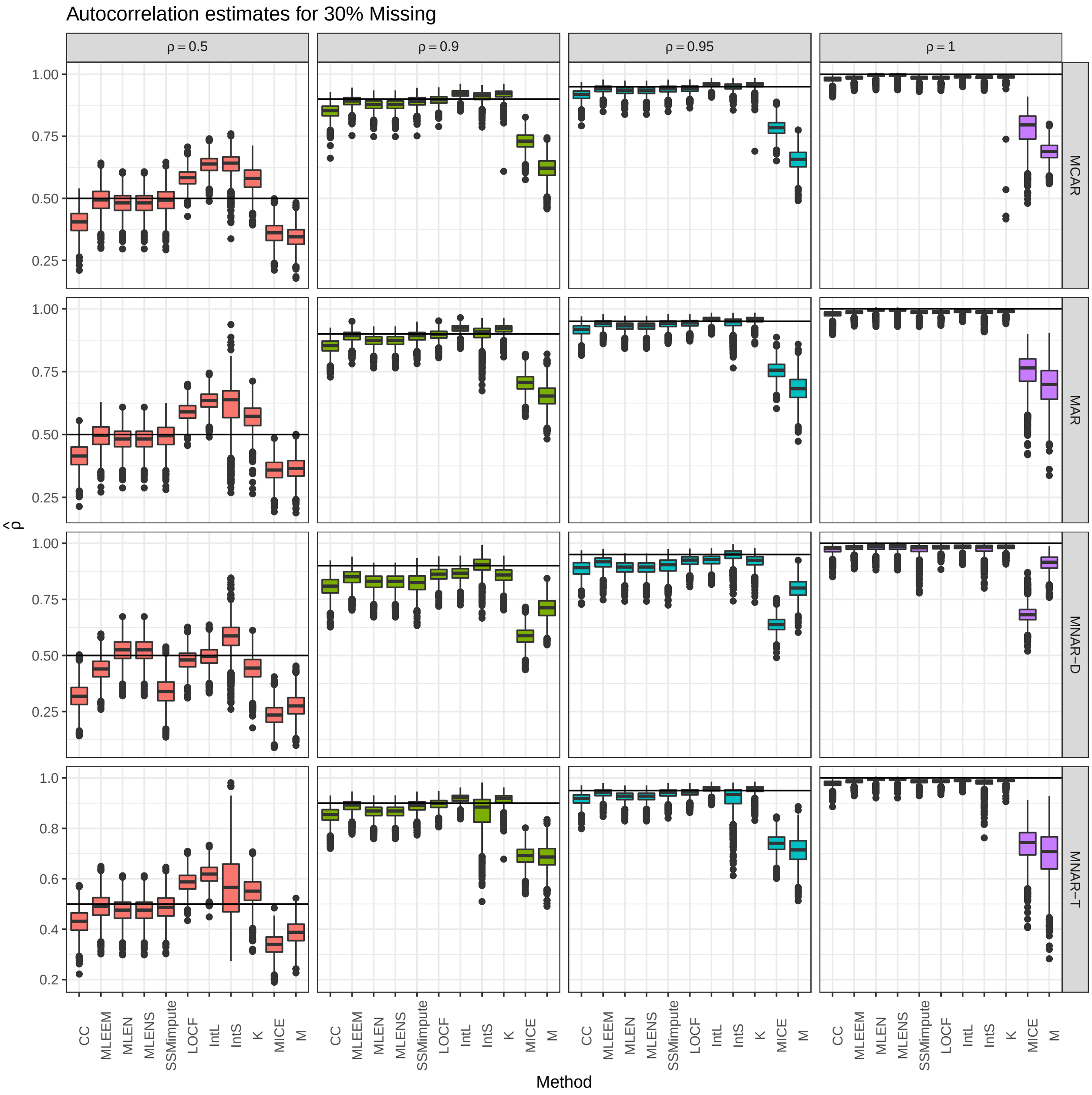

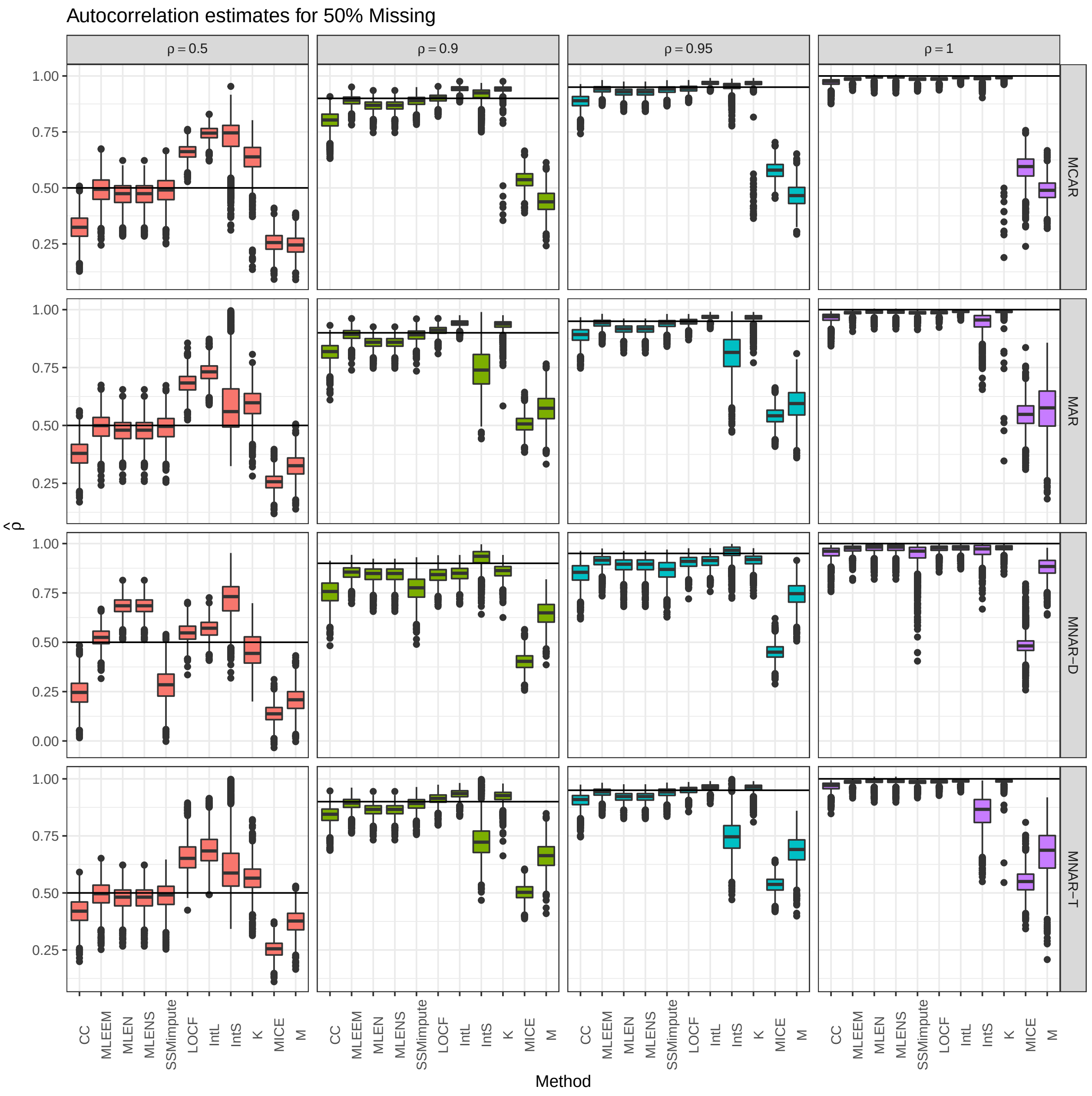

Autocorrelation estimates for 50% Missing
ρ = 0.5
ρ = 0.9
ρ = 0.95
ρ = 1
MCAR
MAR
MNAR−D
MNAR−T
0.00
0.25
0.50
0.75
1.00
ρ̂
CC
MLEEM
MLEN
MLENS
SSMimpute
LOCF
IntL
IntS
K
MICE
M
Method

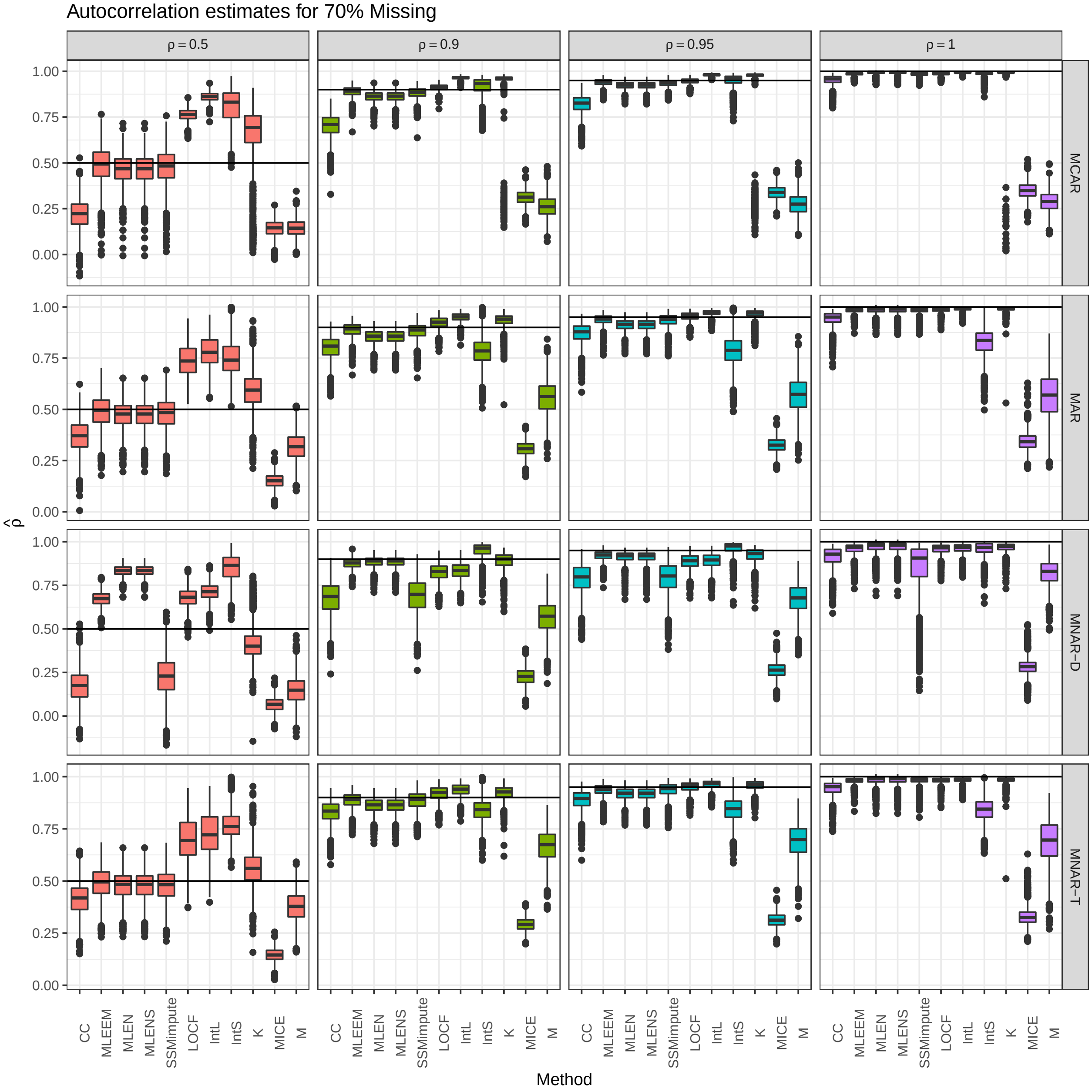
Autocorrelation estimates for 70% Missing
ρ = 0.5
ρ = 0.9
ρ = 0.95
ρ = 1
MCAR
MAR
MNAR−D
MNAR−T
ρ̂
1.00
0.75
0.50
0.25
0.00
CC
MLEEM
MLEN
MLENS
SSMimpute
LOCF
IntL
IntS
K
MICE
M
Method

“ ‘
