# Supplementary material for: Testing unit root non-stationarity in the presence of missing data in univariate time series of mobile health studies
Source: J R Stat Soc Ser C Appl Stat. 2024 Feb 29;73(3):755–73. doi: 10.1093/jrsssc/qlae010 (PMC11175825; doi:10.1093/jrsssc/qlae010)
Supplement: qlae010_Supplementary_Data [file qlae010_supplementary_data.zip › Web Appendix B.pdf]

Web Appendix B: Sensitivity Analysis Simulation Results

P-Value Visualization

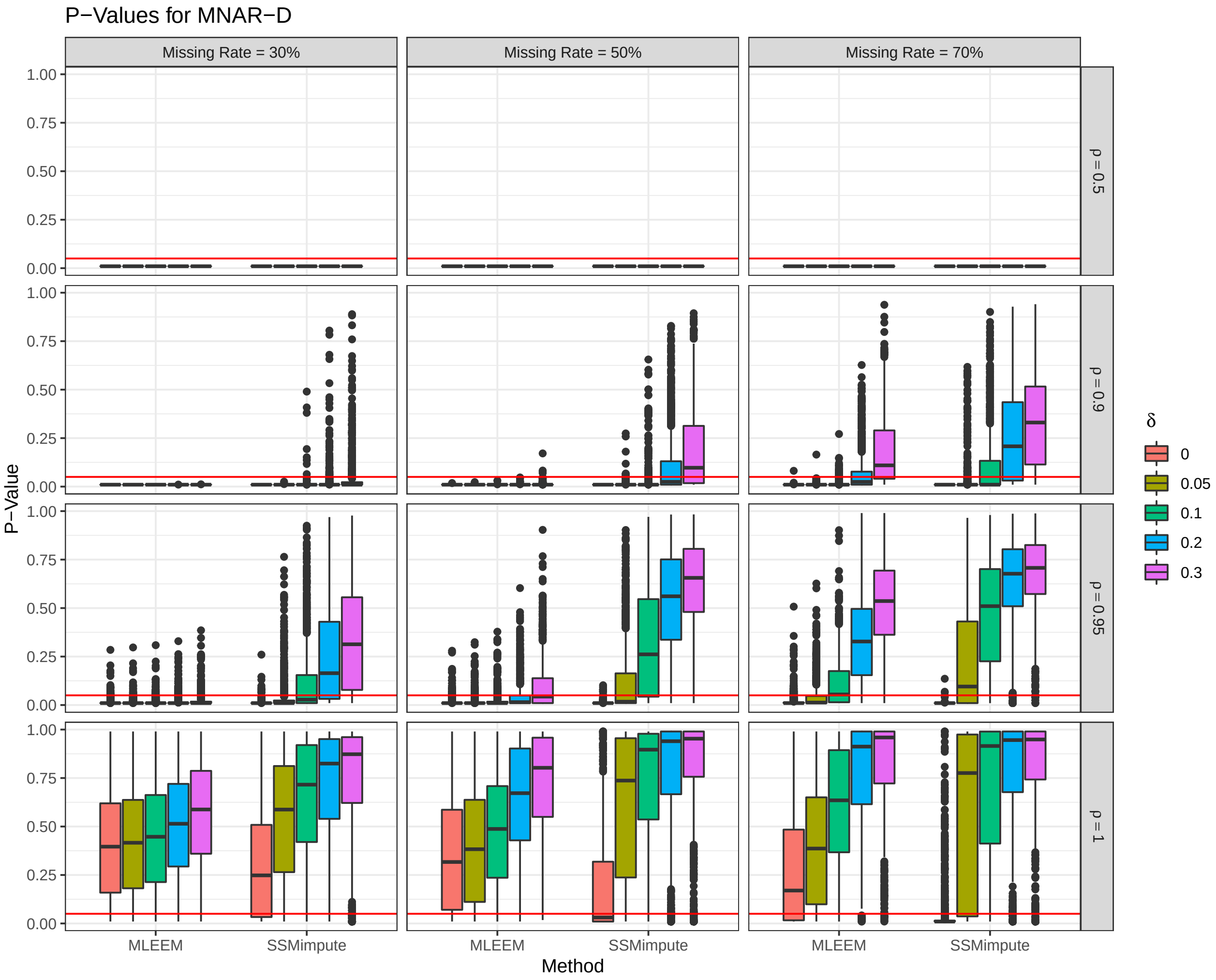

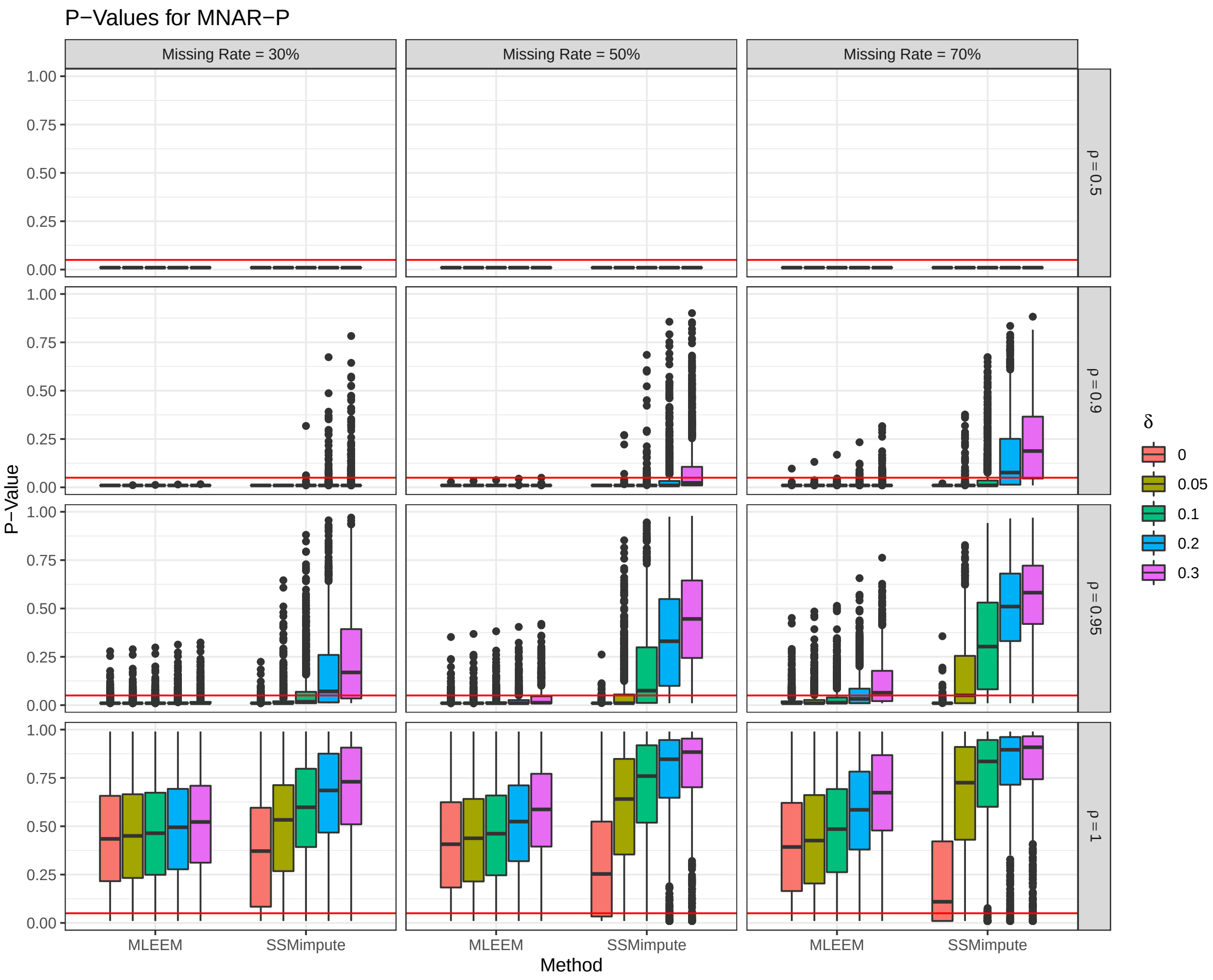
P−Values for MNAR−P
Missing Rate = 30%
Missing Rate = 50%
Missing Rate = 70%
ρ = 0.5
ρ = 0.9
ρ = 0.95
ρ = 1
1.00
0.75
0.50
0.25
0.00
P−Value
MLEEM
SSMimpute
Method
δ
0
0.05
0.1
0.2
0.3

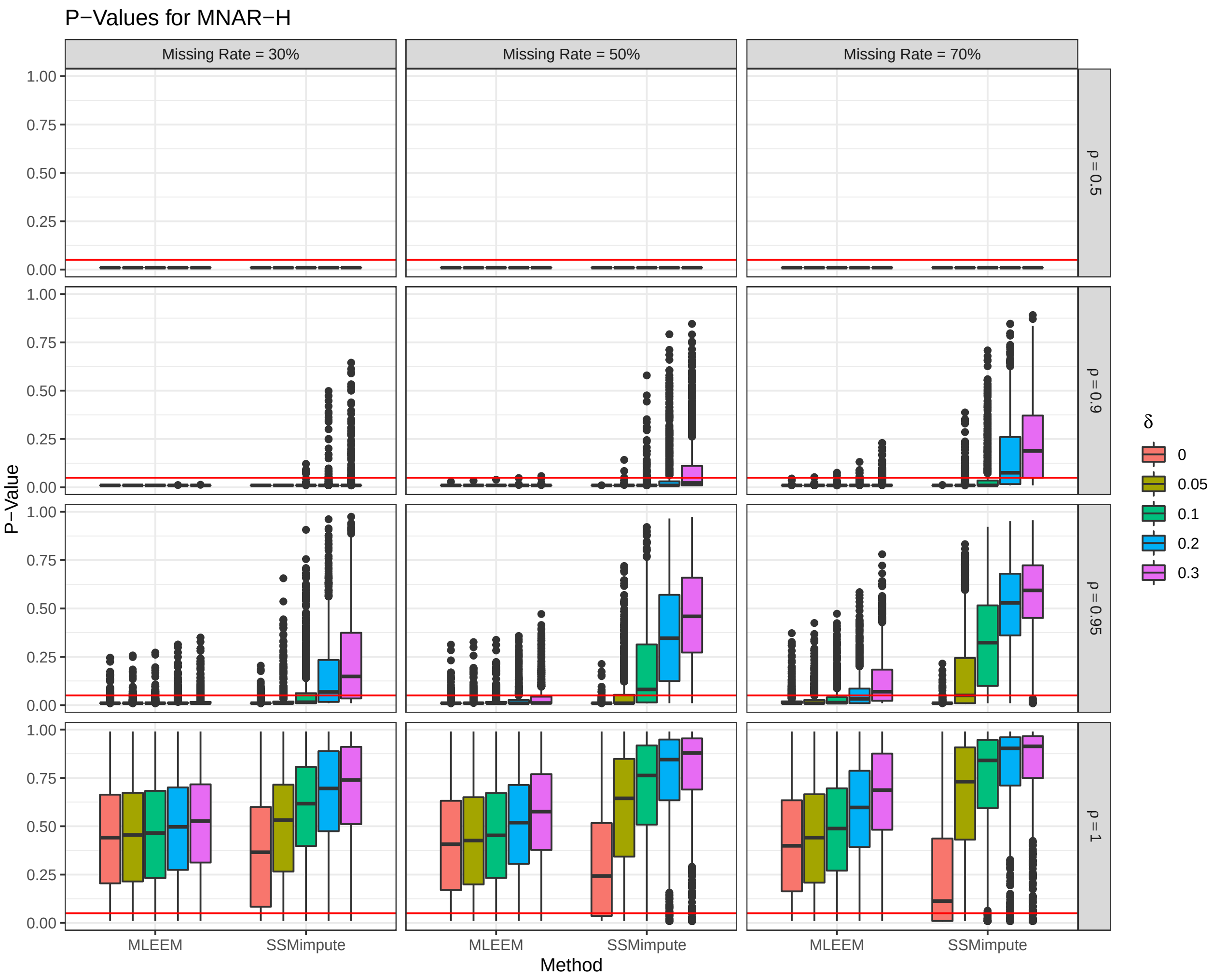

P−Values for MNAR−H
Missing Rate = 30%
Missing Rate = 50%
Missing Rate = 70%
ρ = 0.5
ρ = 0.9
ρ = 0.95
ρ = 1
P−Value
1.00
0.75
0.50
0.25
0.00
MLEEM
SSMimpute
Method
δ
0
0.05
0.1
0.2
0.3

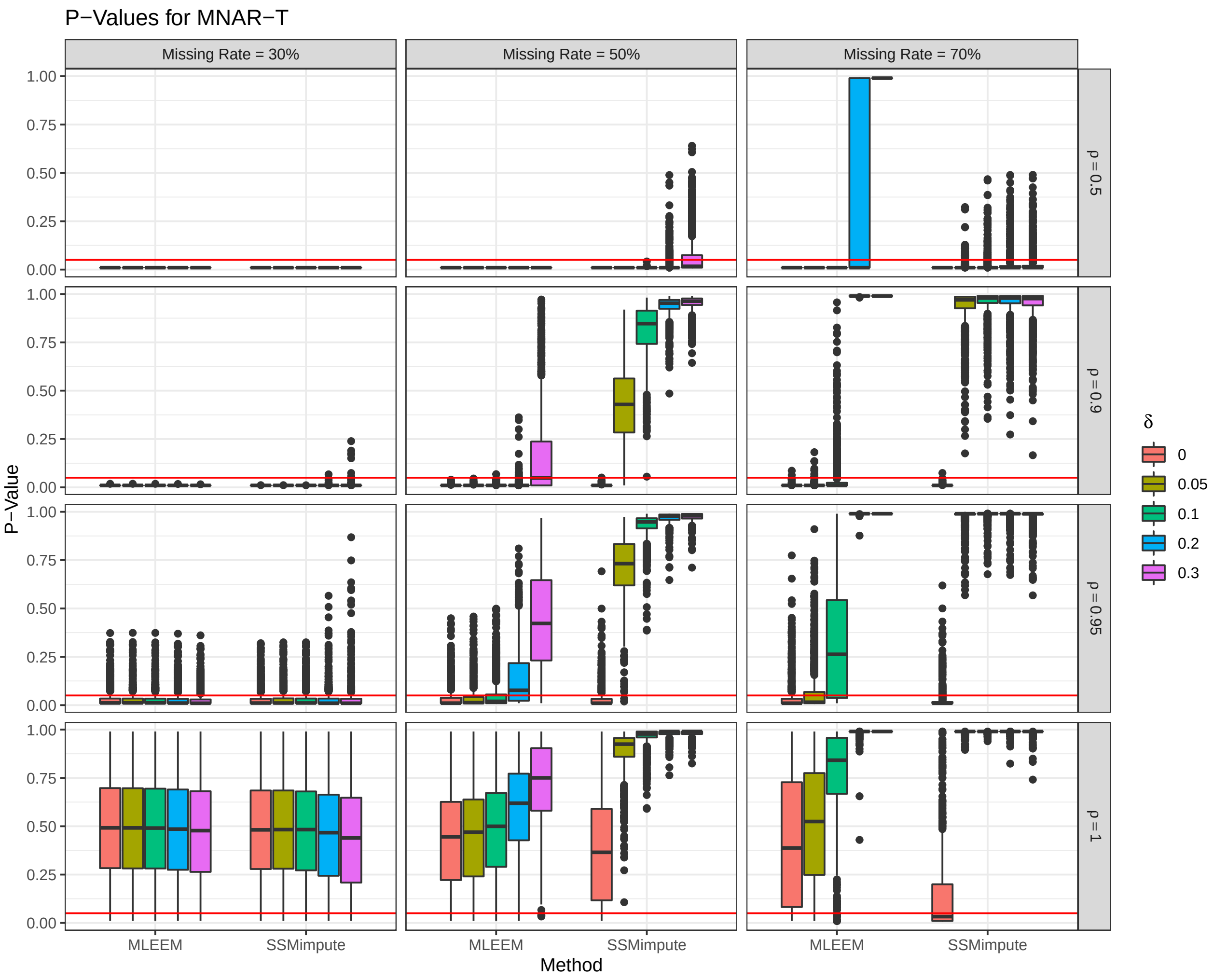
P−Values for MNAR−T
Missing Rate = 30%
Missing Rate = 50%
Missing Rate = 70%
ρ = 0.5
ρ = 0.9
ρ = 0.95
ρ = 1
P−Value
1.00
0.75
0.50
0.25
0.00
MLEEM
SSMimpute
Method
δ
0
0.05
0.1
0.2
0.3

Autocorrelation Vizualization

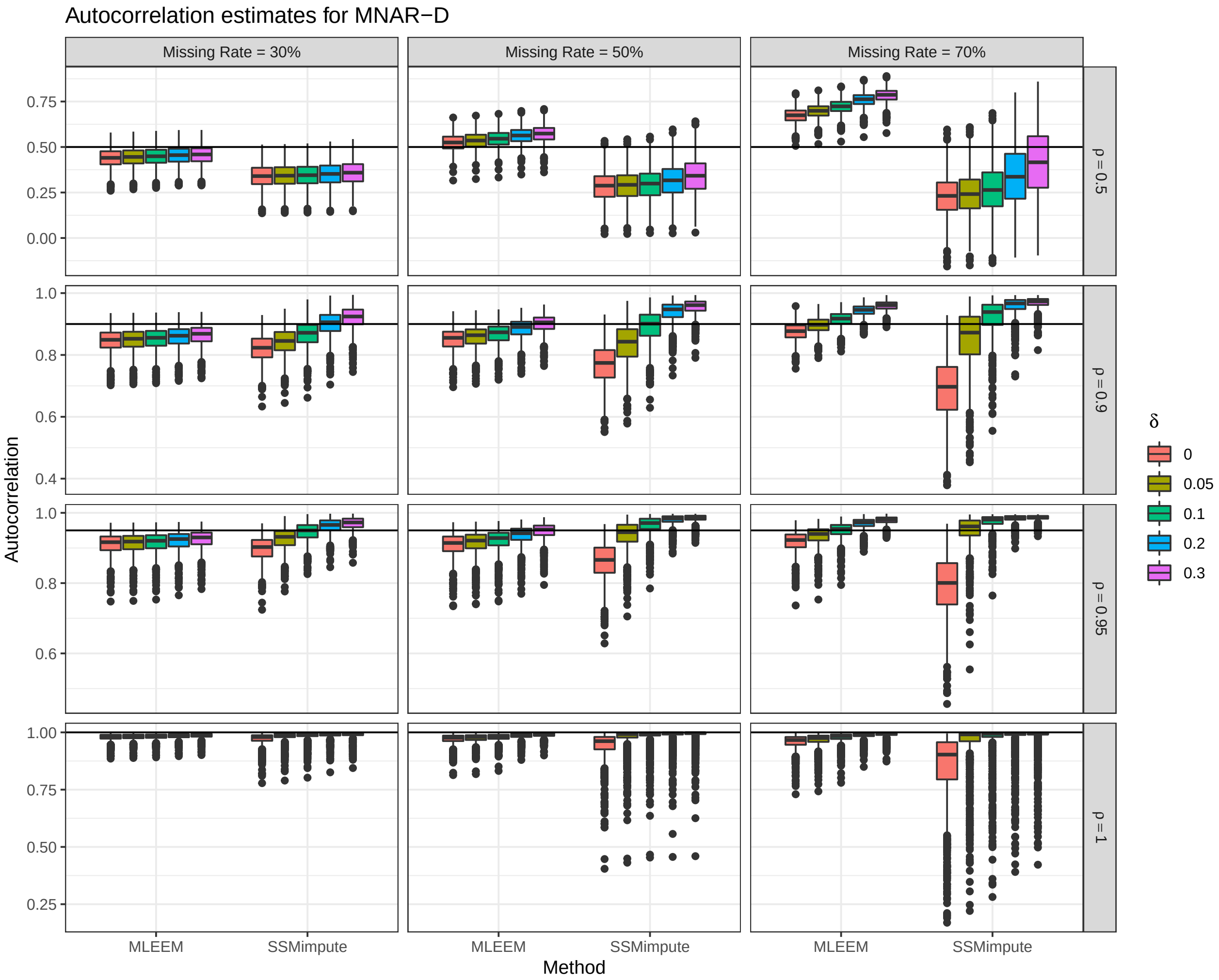

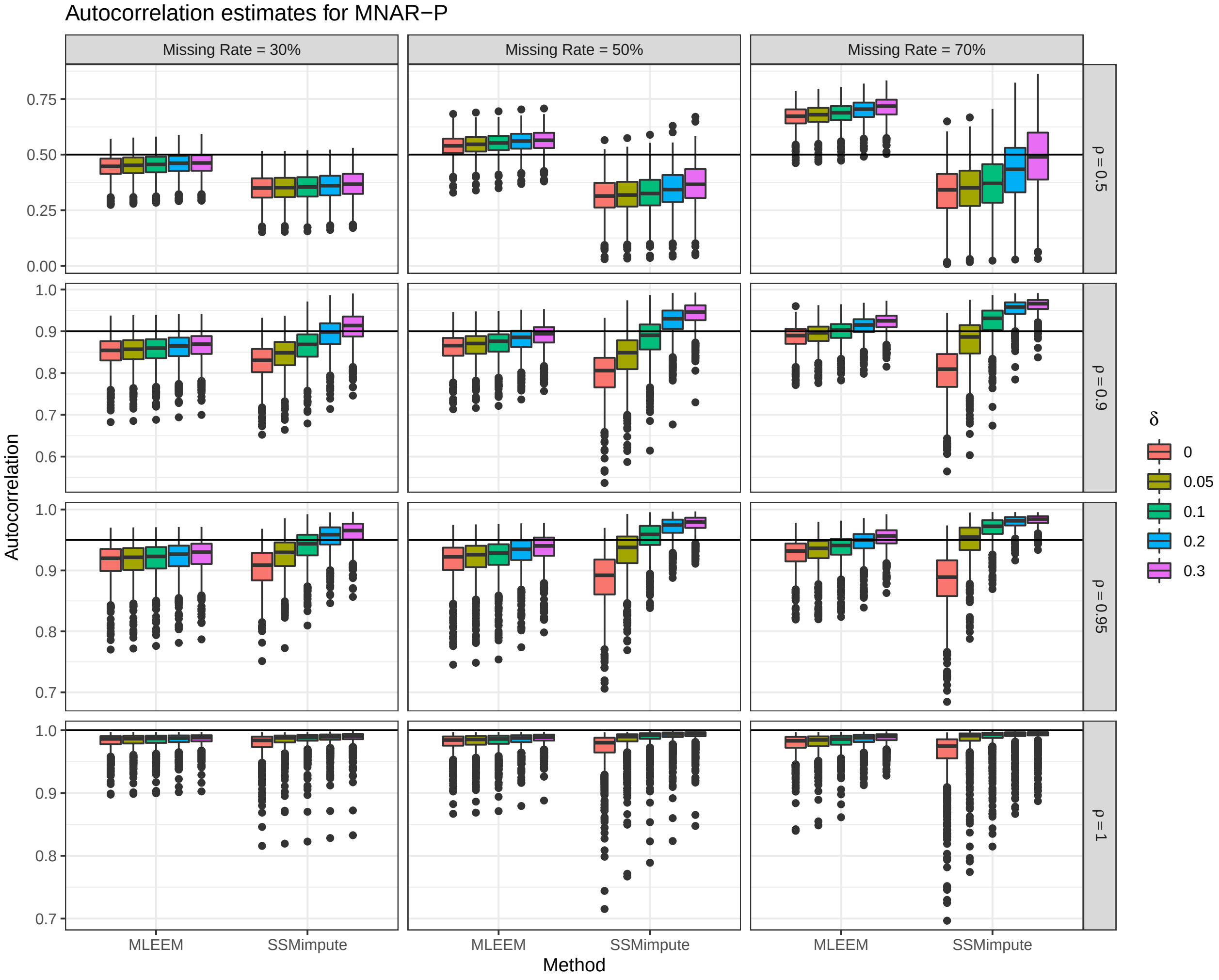

Autocorrelation estimates for MNAR−P
Missing Rate = 30%
Missing Rate = 50%
Missing Rate = 70%
ρ = 0.5
ρ = 0.9
ρ = 0.95
ρ = 1
Autocorrelation
Method
MLEEM
SSMimpute
δ
0
0.05
0.1
0.2
0.3

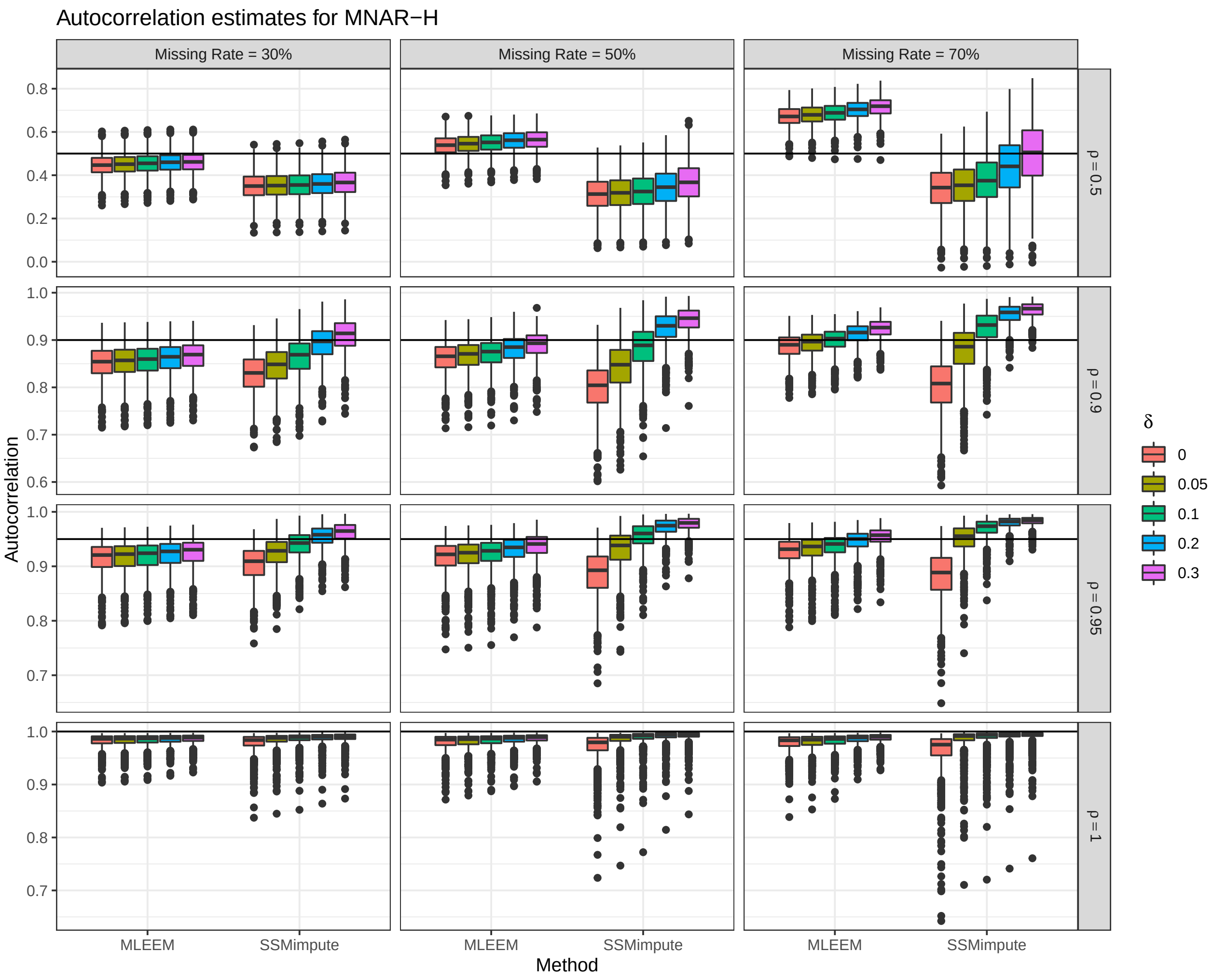

Autocorrelation estimates for MNAR−H
Missing Rate = 30%
Missing Rate = 50%
Missing Rate = 70%
ρ = 0.5
ρ = 0.9
ρ = 0.95
ρ = 1
Autocorrelation
Method
MLEEM
SSMimpute
δ
0
0.05
0.1
0.2
0.3

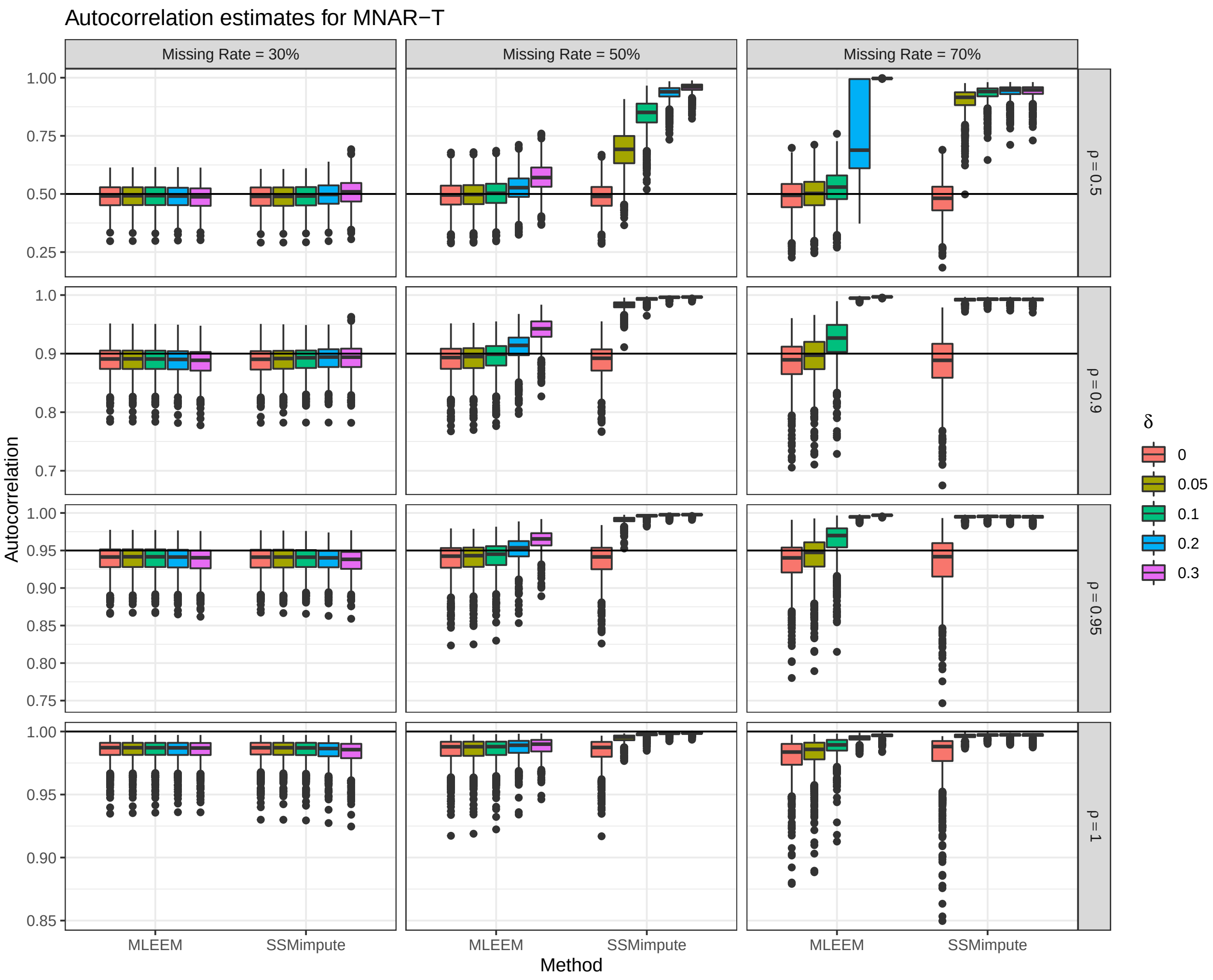
Autocorrelation estimates for MNAR−T
Missing Rate = 30%
Missing Rate = 50%
Missing Rate = 70%
ρ = 0.5
ρ = 0.9
ρ = 0.95
ρ = 1
Autocorrelation
Method
MLEEM
SSMimpute
δ
0
0.05
0.1
0.2
0.3
