# Supplementary material for: Testing unit root non-stationarity in the presence of missing data in univariate time series of mobile health studies
Source: J R Stat Soc Ser C Appl Stat. 2024 Feb 29;73(3):755–73. doi: 10.1093/jrsssc/qlae010 (PMC11175825; doi:10.1093/jrsssc/qlae010)
Supplement: qlae010_Supplementary_Data [file qlae010_supplementary_data.zip › Web Appendix C.pdf]

Web Appendix C: Simulation Results with non-normal errors

Results are from 500 simulations with t-distributed errors (df = 5).

P-Value Visualization

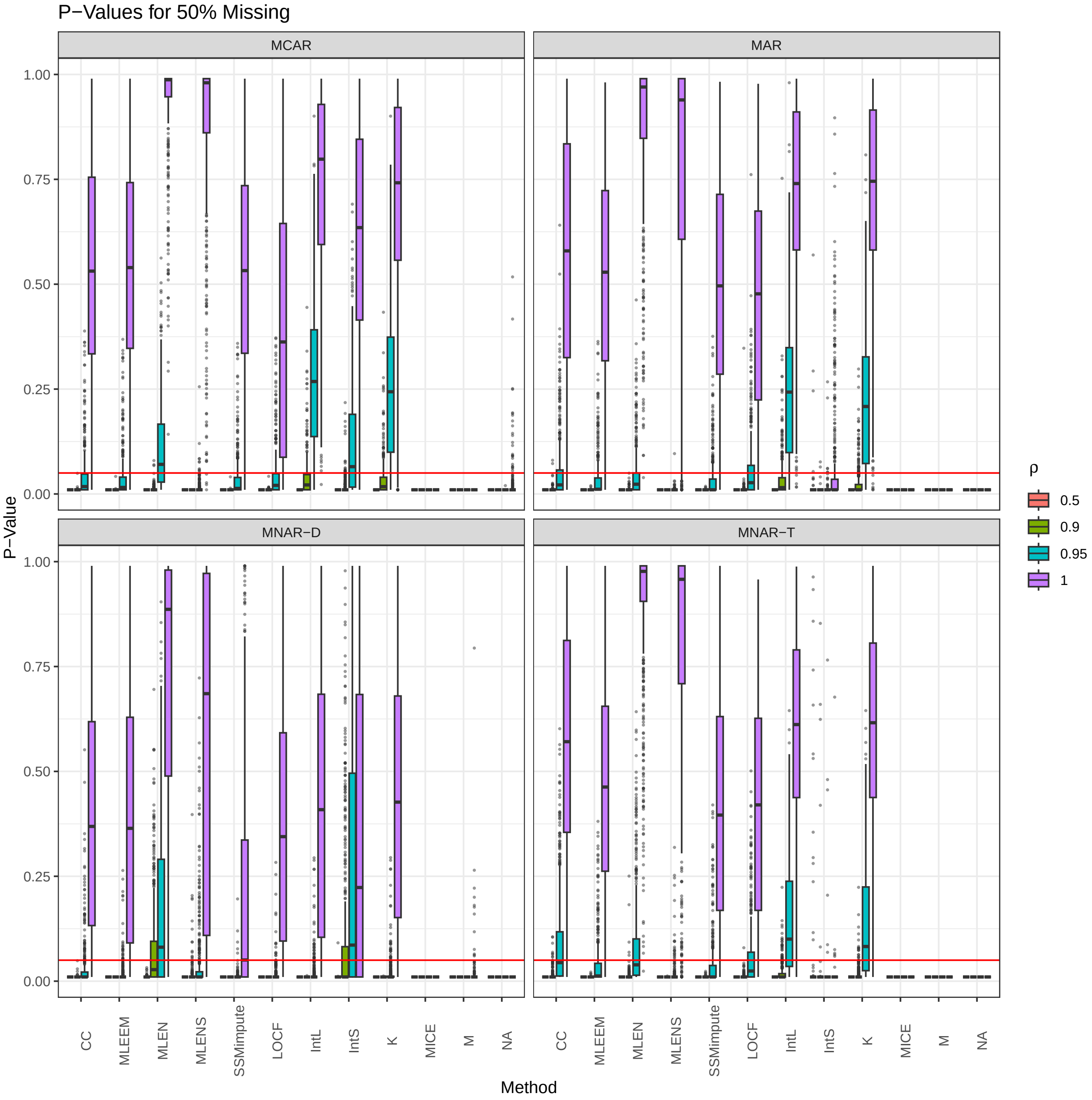

Test Statistic Visualization

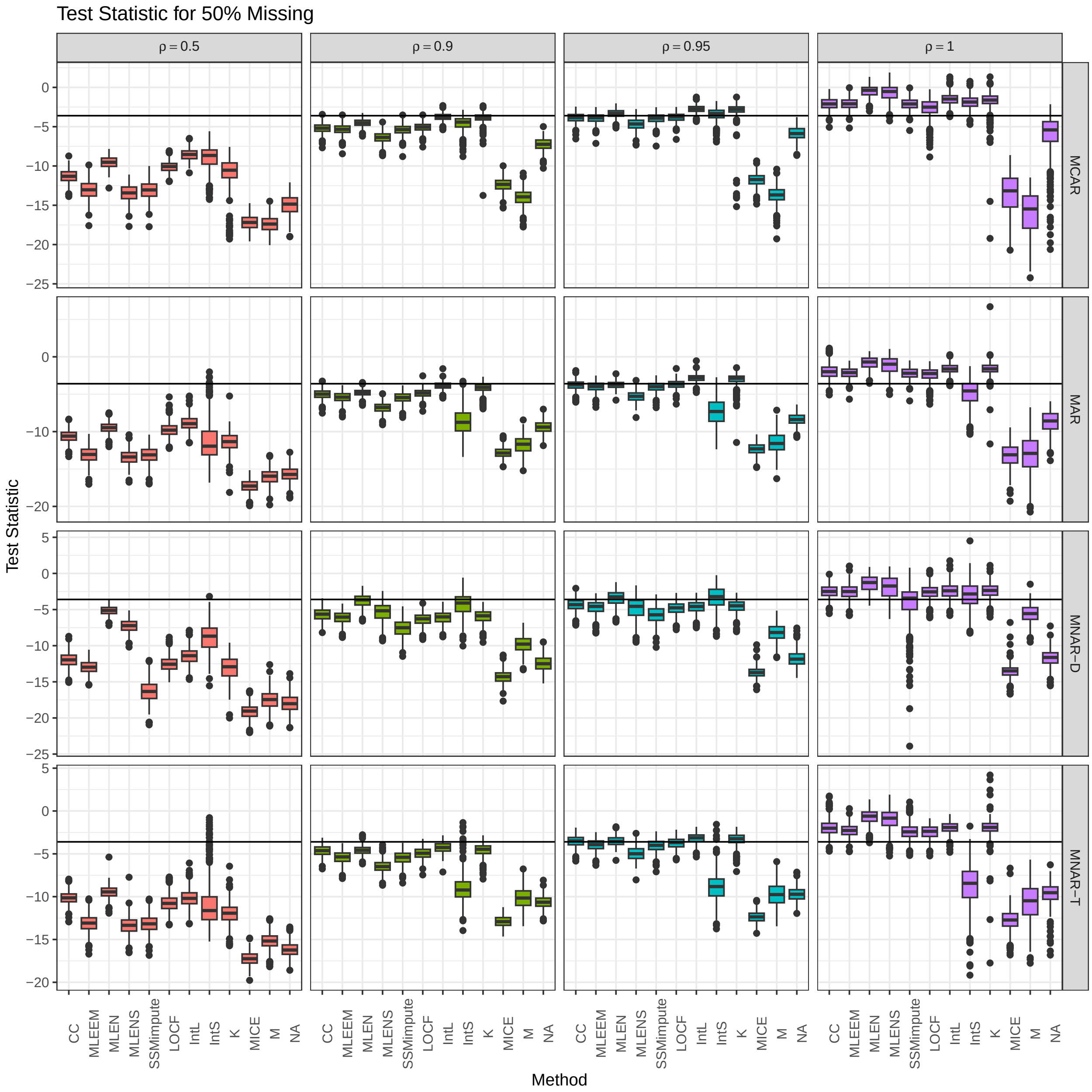

Autocorrelation Vizualization

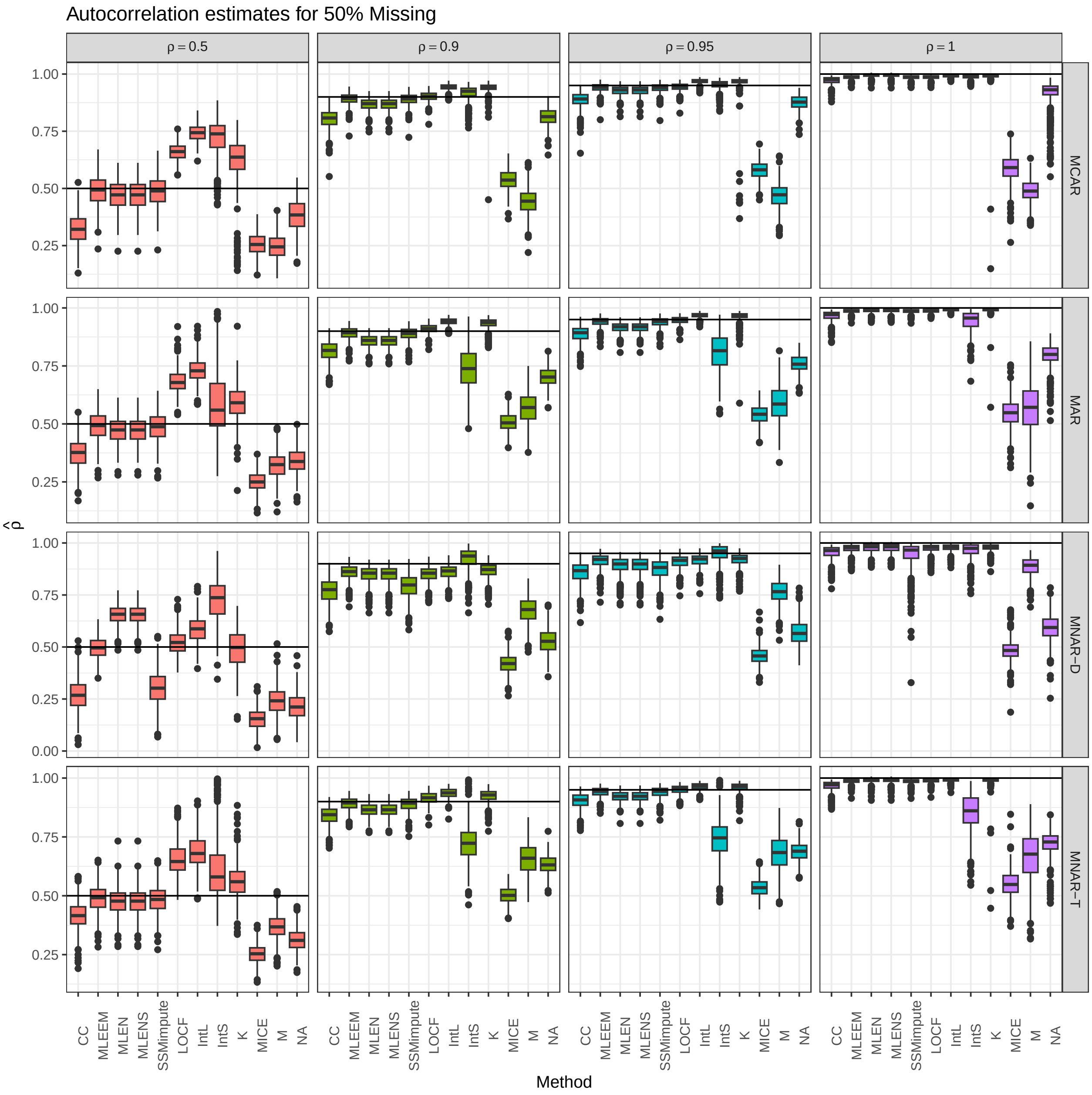

"'
